# Effects of annealing temperature and duration on the morphological and optical evolution of self-assembled Pt nanostructures on c-plane sapphire

**DOI:** 10.1371/journal.pone.0177048

**Published:** 2017-05-04

**Authors:** Mao Sui, Ming-Yu Li, Sundar Kunwar, Puran Pandey, Quanzhen Zhang, Jihoon Lee

**Affiliations:** 1 College of Electronics and Information, Kwangwoon University, Nowon-gu Seoul, South Korea; 2 Institute of Nanoscale Science and Engineering, University of Arkansas, Fayetteville, AR, United States of America; Institute of Materials Science, GERMANY

## Abstract

Metallic nanostructures (NSs) have been widely adapted in various applications and their physical, chemical, optical and catalytic properties are strongly dependent on their surface morphologies. In this work, the morphological and optical evolution of self-assembled Pt nanostructures on *c*-plane sapphire (0001) is demonstrated by the control of annealing temperature and dwelling duration with the distinct thickness of Pt films. The formation of Pt NSs is led by the surface diffusion, agglomeration and surface and interface energy minimization of Pt thin films, which relies on the growth parameters such as system temperature, film thickness and annealing duration. The Pt layer of 10 nm shows the formation of overlaying NPs below 650°C and isolated Pt nanoparticles above 700°C based on the enhanced surface diffusion and Volmer-Weber growth model whereas larger wiggly nanostructures are formed with 20 nm thick Pt layers based on the coalescence growth model. The morphologies of Pt nanostructures demonstrate a sharp distinction depending on the growth parameters applied. By the control of dwelling duration, the gradual transition from dense Pt nanoparticles to networks-like and large clusters is observed as correlated to the Rayleigh instability and Ostwald ripening. The various Pt NSs show a significant distinction in the reflectance spectra depending on the morphology evolution: i.e. the enhancement in UV-visible and NIR regions and the related optical properties are discussed in conjunction with the Pt NSs morphology and the surface coverage.

## Introduction

In last decade, the Pt nanostructures (NSs) have been adapted in widespread applications such as electro-catalytic systems [1–3], hydrogen storages [4, 5], light emitting diodes (LEDs) [6], solar cells [7] and photocatalytic applications [8, 9]. For example, as a catalyst of high activity and selectivity, the Pt NSs utilized in the electro-chemical applications such as fuel cells can significantly improve the reaction efficiency for the oxidation and oxygen reduction benefitted by the enlarged surface area to volume ratio [1–3]. In addition, the Pt NSs can be utilized to improve the hydrogen storage capacity in the carbon, zeolites and metal-organic based spillover hydrogen storages by dissociating hydrogen molecules onto receptors [4, 5]. Recently, due to the localized surface plasmon resonance (LSPR), metallic NSs are reported in various photochemical and optoelectronic applications and their size, density and configuration dependent light interactions can be utilized to tailor the optical properties, e.g. spectral absorption and scattering [10–12]. Unlike the widely studied Ag and Au NSs with well-defined LSPR absorption band, the Pt NSs exhibit a more broadened light absorption in the ultraviolent-visible regions. Profited by this, the performance of Pt NSs can potentially exceed the Ag and Au NSs in specific cases. For instance, the Pt catalyzed aerobic oxidation of alcohols has demonstrated a quantum yield of 7.1% under visible light irradiation due to the significantly promoted photo-catalysis activity, which is nearly twice the case of Au NS photo-catalysts [8, 9]. Also, an over 57% output power increase is reported in an LSPR-enhanced ultraviolet light-emitting diode with the employment of Pt NSs, sharply increased as compared with the Ag NS case of 20% [6]. The LSPR properties can be adequately tuned by the control of NS configuration, size and density, therefore, a vital requirement is raised for the controlled Pt NS fabrication [13–17]. As an effective approach of self-assembly through physical vapor deposition (PVD), the solid state dewetting (SSD) can achieve a precisely modulated NS fabrication on the substrate and thus has been reported in the controllable fabrication of various metal NSs, e.g. Au, Ag and Rh [16, 18–20]. Meanwhile, sapphire with the high thermal durability, mechanical strength and chemical stability has widely adapted in electronic and optoelectronic devices [21–25]. The controllable fabrication of various Pt NSs on sapphire can provide the essential reference on the pertinent modulation of surface physical, chemical, optical and catalytic properties necessary for the corresponding applications based upon. Therefore, in this work, we study the self-assembled Pt NSs evolution on *c*-plane sapphire (0001) based on the control of annealing temperature and duration, in which the coherent effect of surface diffusion, surface energy minimization, Rayleigh instability and Ostwald ripening lead to the formation of various configuration, size and density of Pt NSs. Based on the morphological and elemental characterizations by AFM, SEM and EDS, the evolution of various Pt NSs are demonstrated and the growth models are discussed with the distinct deposition amount. Furthermore, the optical properties of various Pt NSs are investigated by the reflectance spectra and the effect on lattice vibration is probed by Raman spectra. The corresponding variation of intensity and peak positions are discussed along with the NS morphology as well as the surface coverage changes.

## Materials and methods

### Preparation of substrate and Pt nanostructure fabrication

The Pt NSs were fabricated on *c*-plane sapphire (0001) wafers with a thickness of 430 μm and off-axis ± 0.1 (iNexus Inc., South Korea). Initially, the wafers were diced into 6 × 6 mm^2^ squares using a machine saw. In order to prepare the substrate for the deposition, each sample was degassed at 650°C for 900 s under a vacuum below 1 × 10^−4^ Torr. During the degassing, an Inconel blank was mounted on the backside of sample to establish a stable thermal conduction from a halogen lamp. After the degassing, the sapphire surface showed a smooth morphology with the height distribution of ± 0.5 nm as shown in S1 Fig. In addition, the Raman spectra of bare sapphire are shown in S1 Fig. Right after the degassing, the Pt layers were deposited in a sputter chamber with a growth rate of 0.05 nm/s and ionization current of 3 mA under 1 × 10^−1^ Torr at ambient temperature. The temperature effect on the Pt NS fabrication was investigated with three distinct deposition thickness, i.e. 3, 10 and 20 nm, which were separately deposited by the control of sputtering durations. After the Pt film deposition, the samples showed uniform surface morphologies and the surface roughness was slightly increased along with the Pt thickness as shown in S2 Fig. Subsequently, the samples were transferred to a pulsed laser deposition (PLD) chamber for annealing at various temperature between 500 and 950°C with a constant ramping rate of 4°C/s under 1 × 10^−4^ Torr. After reaching the target temperature, a dwelling duration of 450 s was equally applied to each fabrication to guarantee a sufficient surface diffusion. The effect of annealing duration was investigated between 0 and 3600 s at 800°C with the Pt deposition thickness of 15 and 20 nm. Meanwhile, the ramping rate was accelerated to 10°C/s in order to minimize the ripening effect during the temperature-rise process [26, 27]. After each growth, the substrate temperature was immediately quenched down to the ambient to eliminate the undesired ripening.

### Characterization of Pt nanostructures

After the fabrication of Pt NSs, morphological, elemental and optical characterizations were carried out. An atomic force microscope (AFM) (Park Systems, South Korea) non-contact mode was utilized to obtain the three dimensional surface topography and a scanning electron microscope (SEM) (CoXEM, South Korea) was employed for the larger scale 2D images. The AFM tips (NSC16/AIBS, μmasch, USA) utilized were 17–21 μm in length with a curvature radius less than 10 nm. The spring constant and resonant frequency of cantilevers were ~ 40 N/m and ~ 300 kHz respectively. The original data from AFM was processed by the XEI software (Park Systems) to obtain the top- and side-views, cross-sectional line-profiles, RMS surface roughness (R_q_) and surface area ratio (SAR). The R_q_ and SAR are given by $\sqrt{\frac{1}{n}\sum_{i=1}^{n}y_{i}^{2}}$ and $\frac{\left(A_{G}-A_{S}\right)}{A_{G}}\times 100\;[\%]$ where, the *y*_*i*_ is height at each pixel, A_S_ is surface area (2D) and A_G_ is geometric area (3D). The elemental analysis was performed by an energy-dispersive X-ray spectroscope (EDS) system (Thermo Fisher Noran System 7, USA). The reflectance and Raman spectra were acquired by the UNIRAM II system (UniNanoTech Co. Ltd., South Korea). The light source for reflectance measurement was the combination of deuterium (250 ≤ λ ≤ 450 nm) and halogen (450 ≤ λ ≤ 1100 nm) lamps and the Raman signal was excited by a 532 nm laser at ambient temperature.

## Results and discussion

**Fig 1:**
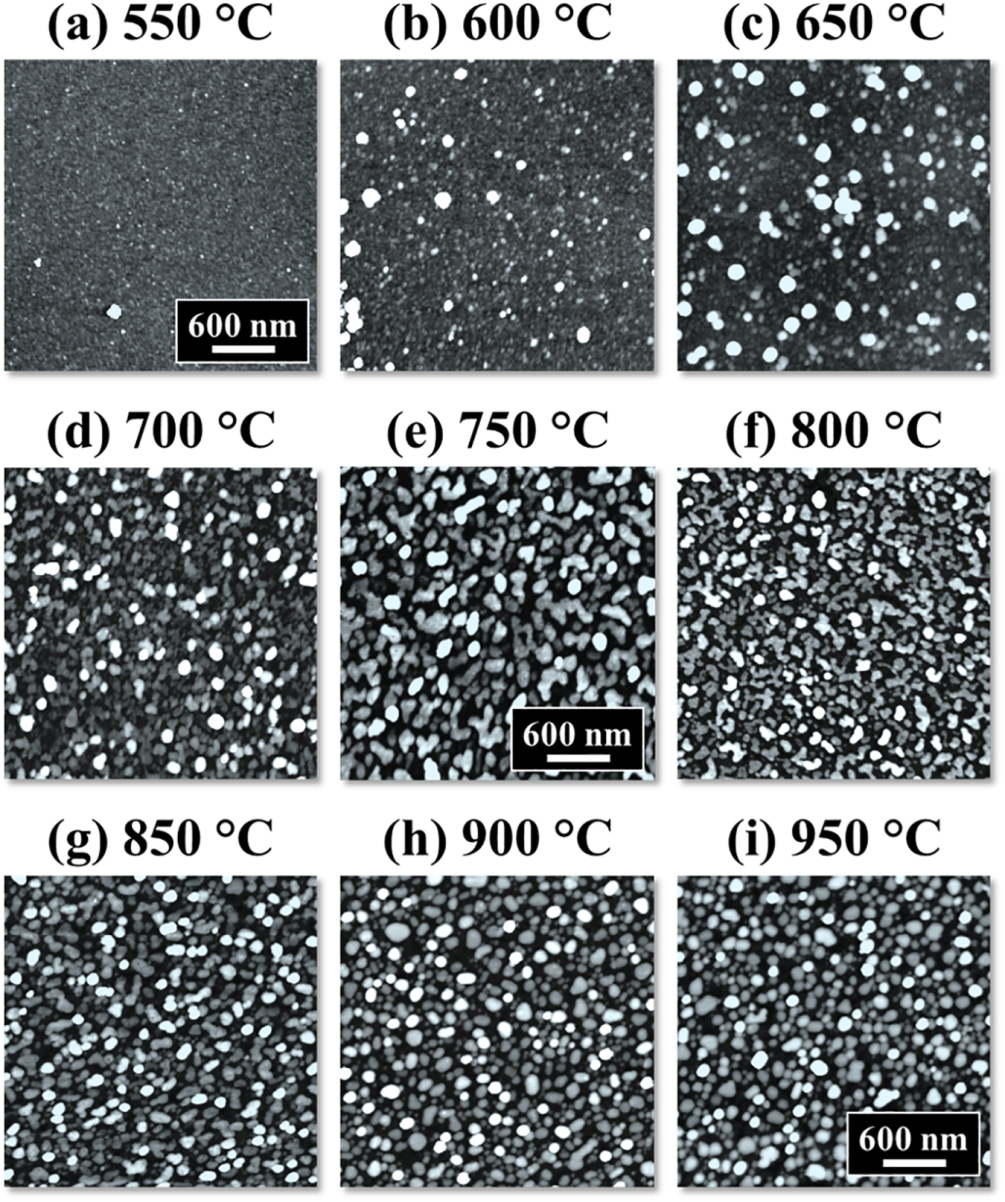
Annealing temperature effect on the evolution of self-assembled Pt nanoparticles (NPs) on sapphire (0001). Each sample was deposited with 10 nm thick Pt layer and annealed between 550 and 950°C for the identical duration of 450 s. (a)—(i) Atomic force microscope (AFM) top-views of 3 × 3 μm^2^.

**Fig 2:**
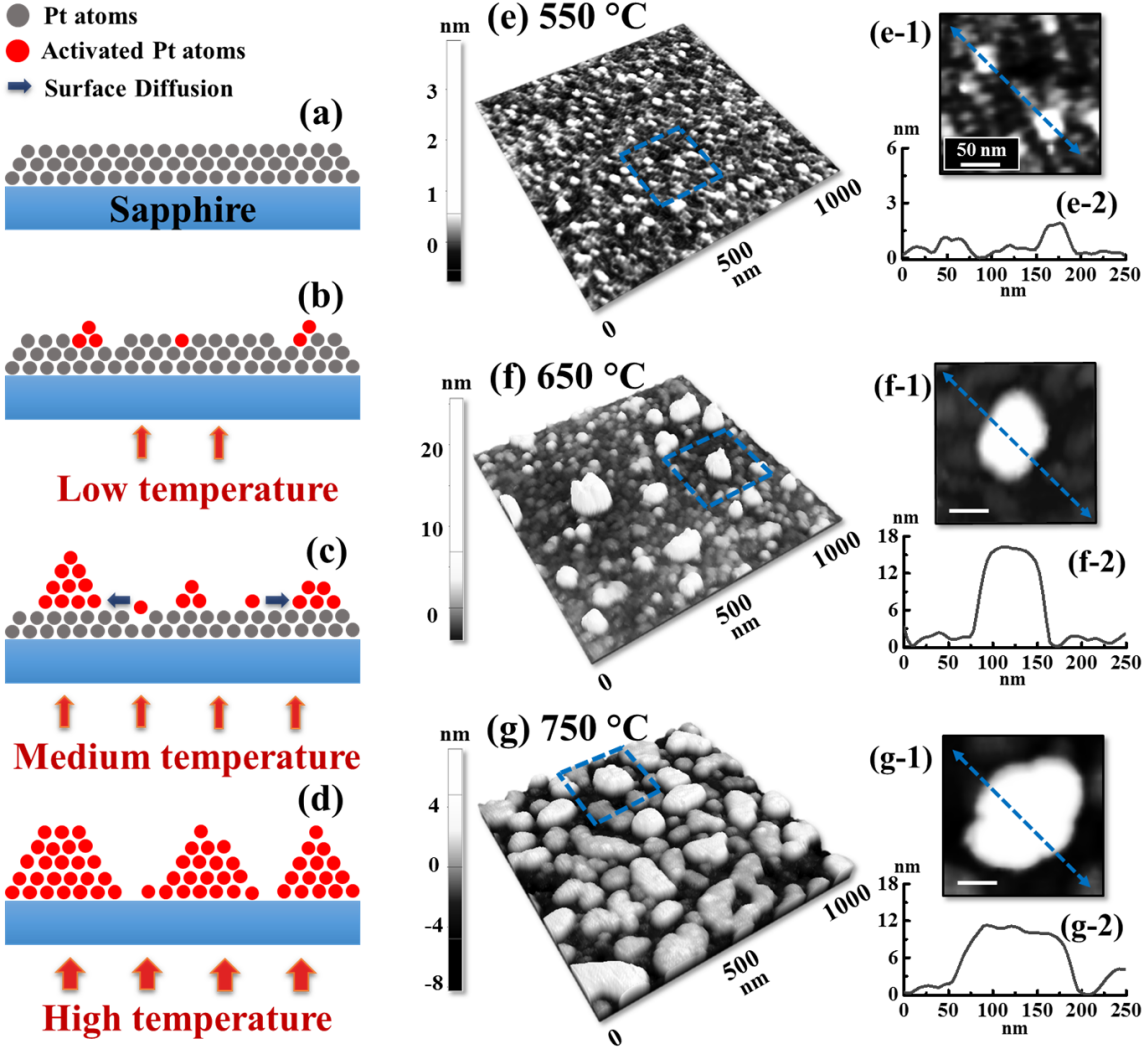
Pt NPs nucleation and growth at lower annealing temperature range between 550 and 750°C with the 10 nm initial film thickness and 450 s annealing duration. (a)–(d) Schematics of diffusion process depending on temperature. (e)–(g) AFM side-views of 1 × 1 μm^2^ and top-views of 200 × 200 nm^2^ in (e-1)–(g-1). (e-2)–(g-2) Cross-sectional line-profiles acquired from the locations indicated by the blue lines in (e-1)–(g-1).

**Fig 3:**
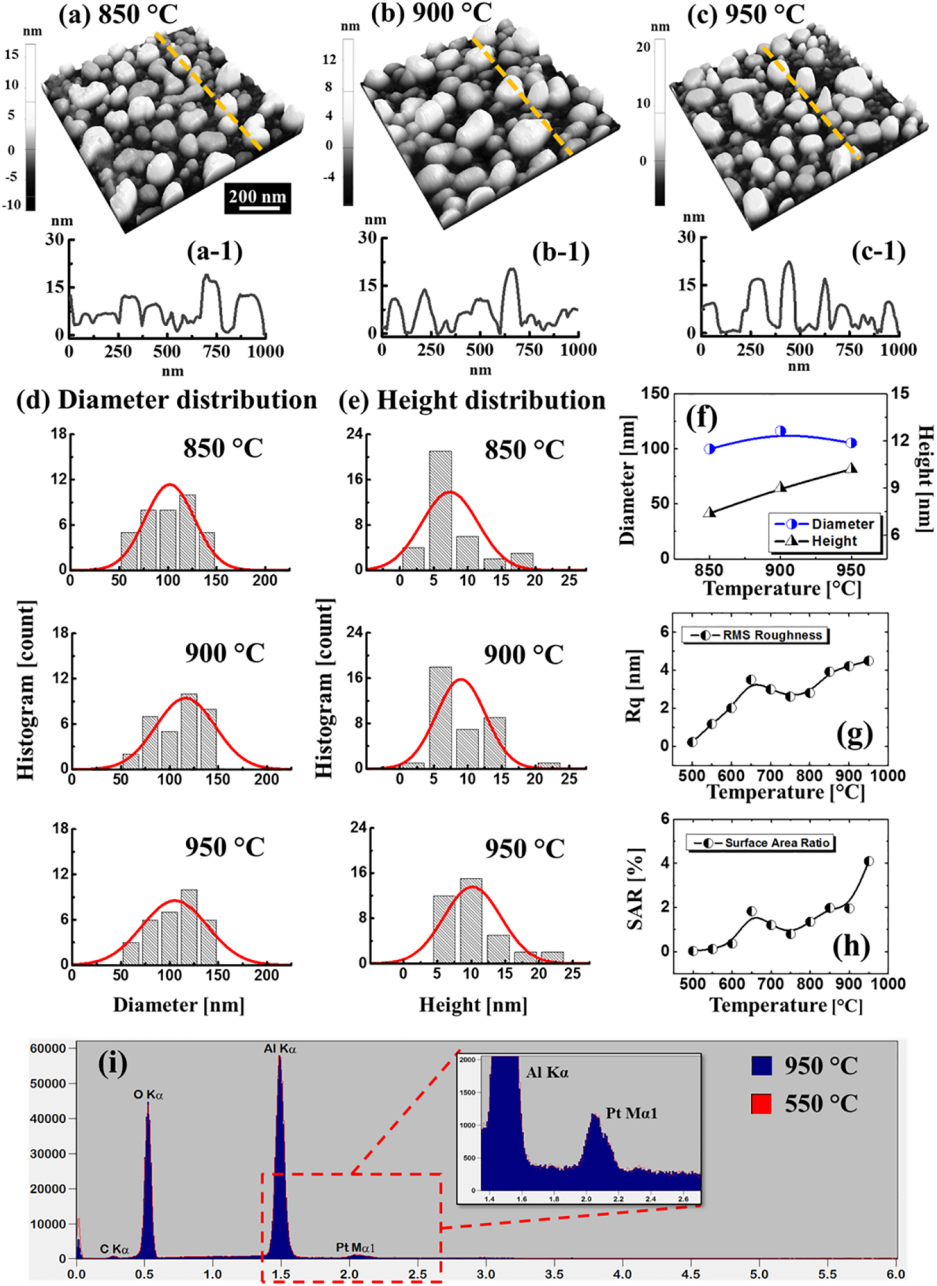
Evolution of Pt NPs on c-plane sapphire between 850 and 950°C with the 10 nm thickness. (a)–(c) AFM side-views of 1 × 1 μm^2^. (a-1)–(c-1) Corresponding line-profiles. (d) Diameter and (e) height distribution histograms. Summary plots of (f) diameter and height, (g) root mean squared roughness (Rq) and (h) surface area ratio (SAR) along with the annealing temperature. Error range: ± 5% in (f). (i) Energy dispersive x-ray spectroscope (EDS) elemental characterization of 550 and 950°C samples in the 10 nm set, showing well matched spectra. (Inset) Enlarged range between 1.4 and 2.6 keV depicts Pt Mα1 peak.

**Fig 4:**
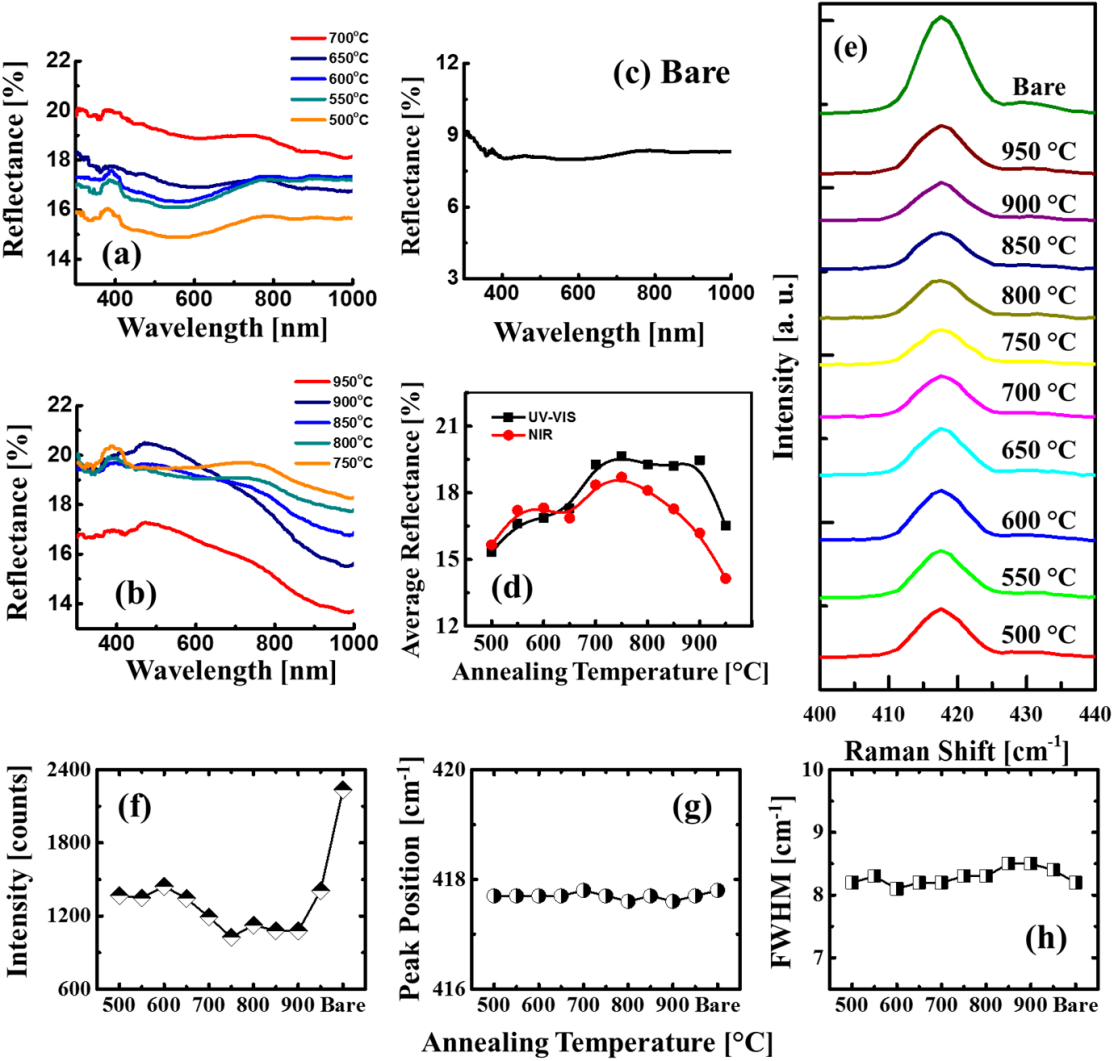
Reflectance and Raman spectra of the Pt NPs on sapphire (0001) fabricated between 500 and 950°C with the 10 nm film thickness. (a)–(b) UV-VIS-NIR reflectance spectra of corresponding samples. (c) Reflectance spectrum of bare sapphire. (d) Average reflectance plotted with respect to the annealing temperature. (e) Corresponding Raman spectra of the A_1g_ peaks. Summary plots of (f) peak intensity, (g) peak position and (h) FWHM.

Fig 1 shows the morphological evolution of self-assembled Pt nanostructures (NSs) on *c*-plane sapphire by the variation of annealing temperature (AT) between 550 and 950°C with the Pt thickness of 10 nm. The detailed morphological and optical characterizations are presented in Figs 2–4 and the corresponding AFM side-views of 3 × 3 μm^2^ are provided in S3 Fig. Generally, through the gradually enhanced surface diffusion by the thermal energy, the formation of Pt nanoclusters first appears on top of the Pt layers and then the isolated Pt nanoparticles (NPs) are gradually formed from the continuous Pt layer along with the increased AT. Depending on the AT, the Pt adatom surface diffusion can mainly occur through two typical paths: (i) on the Pt surface at lower AT and (ii) on the sapphire surface at higher AT. The surface diffusion of adatoms can be described by the relation [23]:

$$D_{S}=D_{0}exp\left(-\frac{E}{kT}\right),$$ (1)

where the *D*_0_ is the pre-exponential factor, *k* is Boltzmann constant, *T* is the system temperature and *E* is the activation energy of Pt atoms [28]. From the Eq (1), it can be inffered that the *D*_*S*_ is direclty dependent on the *T*. Thus, for a single Pt adatom, it has a high possibility to be activated and diffuse on the surface at increased AT and the amount of activated Pt atoms can be significantly increased at higher AT from a macro pespective. As described in Fig 2(B), with an increased thermal energy, the topmost adatoms on the Pt layer can be first activated and diffuse on the developing overlaying Pt layer. Subsequently, the collisions among the diffusing atoms can take place and result in the nucleation of tiny nanoclusters at increased AT. As indicated in Fig 2(C), as the diffusion length *l*_*D*_ can be enhanced at an increased temperature [29, 30], the diffusing Pt adatoms can have high possibility to travel a further distance and be adsorbed by the nanoclusters driven by the surface energy minimization mechanism [31, 32]. Finally, at a sufficient high AT, the Pt adatom diffusion and nucleation would directly occur on the sapphire substrate as seen in Fig 2(D), resulting in the formation of Pt islands (NPs) according to the Volmer–Weber growth model [33, 34]. With the stronger binding energy between Pt adatoms (*γ*_*Pt*_) as compared to that between Pt and substrate atoms (*γ*_*I*_), namely *γ*_*Pt*_ > *γ*_*I*_, the Pt adatoms tend to bind with each other and grow in 3-dimensional islands and therefore, the continous Pt layer can gradaully develop into the individual Pt NSs along with the increased AT. As shown by the AFM side- and top-views in Fig 2(E) and 2(E-1), the formation of tiny nanoclusters adopting irregular configuration was observed with high density due to the limited surface diffusion and the height of typical nanoclusters was ~ 2 nm. Then, when the AT was increased to 650°C, the dimension of NSs was obviously increased with the enhanced surface diffusion, i.e. NSs height reached over 15 nm. Then at 750°C, the Pt layer was significantly dewetted by the increased thermal energy, resulting in the formation of isolated NSs on sapphire surface as seen in Fig 2(G). As evidenced by the line profiles in Fig 2(F-2) and 2(G-2), the Pt NSs formed at 750°C showed a preferential lateral growth with the slight decrease in height. The configurational variation can be caused by the surface energy difference between Pt and sapphire. As the Pt NSs formed directly on the sapphire substrate, the dewetting angle *θ* can be correspondingly varied according to the Young’s equation and determined by the varied interfacial energies [35]. Between 750 and 850°C, the nanoclusters were gradually developed into more regular shape through the separation induced by the Rayleigh instability [36, 37]. At the same time, with the enhanced surface diffusion, the surface morphology evolved in terms of height and dimension between 850 and 950°C and showed more preferential vertical growth. As exhibited by the diameter (measured along shorter directions) distribution histogram in Fig 3(D), the median of diameter was first increased from 850 to 900°C, which can be attributed to the further shape development, and the nanoparticle counts of large diameters were clearly increased at 900°C, i.e. above 125 nm. However, at 950°C slight decrease on the nanoparticle diameter was observed as the NSs adopted more packed configuration as seen in Fig 3(C), preferential vertical growth. Meanwhile, in terms of the height, a continuously increasing trend was observed as displayed in Fig 3(E) that depicts the height evolution of NPs. On the other hand, the number of short NPs were significantly decayed along with the increased temperature which can be likely due to the absorption by the large NPs in order to lower the surface free energy. As shown by the summary plots in Fig 3(F), the diameter was 99.9 nm at 850°C, then increased by 1.16 times to 116.3 nm at 900°C and finally decreased by 1.07 times to 105 nm at 950°C. Meanwhile, the height was kept increasing from 7.4 to 10.2 nm by 1.37 times between 850 and 950°C. In addition, the R_q_ and SAR are summarized in Fig 3(G) and 3(H) and specific values are provided in S2 Table. The R_q_ and SAR are given by $\sqrt{\frac{1}{n}\sum_{i=1}^{n}y_{i}^{2}}$ and $\frac{\left(A_{G}-A_{S}\right)}{A_{G}}\times 100\;[\%]$ where, the *y*_*i*_ is height at each pixel, A_S_ is surface area (2D) and A_G_ is geometric area (3D). Between 500 and 650°C, the R_q_ and SAR were gradually increased due to the nucleation and growth of Pt nanoclusters. Between 650 and 750°C, both the R_q_ and SAR gradually decreased due to the preferential lateral growth as mentioned above. Finally, along with the separation and growth of isolated NSs, the R_q_ and SAR were gradually increased again between 750 and 950°C. The energy-dispersive X-ray spectroscopy (EDS) spectra of 950 and 550°C samples are shown in Fig 3(I) and the spectra of other samples are provided in S4 Fig. As seen in the spectra, Kα peaks for C, O and Al were observed at 0.28, 0.53 and 1.49 keV respectively. Meanwhile, the Pt Mα1 peak was observed at 2.05 keV. As evidenced by the inset, similar counts were observed for all samples between 950 and 550°C, indicating the equal Pt contents without sublimation at relatively high temperature.

Fig 4 shows the reflectance and Raman spectra of the Pt NSs on sapphire (0001) fabricated by the variation of AT with the10 nm Pt thickness. The reflectance spectra were analyzed over UV, visible and NIR regions and generally, the fabrication of Pt NSs showed an enhancement on the reflectance intensity as compared to the bare sapphire as shown in Fig 4(A), 4(B) and 4(C). In the meantime, the spectral shape evolution by the formation of shoulders and dips were observed along with the variation of surface morphology. The shoulders and dips can be related to the localized surface plasmon resonance (LSPR) induced by the Pt NSs. The resonance can be generated by various modes, e.g. dipole and quandrupole [38, 39]. As seen in Fig 4(A) and 4(B), a small shoulder was observed in the UV region with a center position at ~ 380 nm, which can be related to the quandrupolar resonance. Meanwhile, the dipolar resonance can be also generated, however, as the light absorption of Pt is weaker and broad as compared with Ag and Au [18, 19, 40], no clear dipolar peaks were directly observed in the reflectance evolution. As seen in Fig 4(A) and 4(B), the reflectance was gradually increased between 500 and 700°C whereas it was gradually attenuated above 750°C. Also, clear distinctions were observed between UV-VIS region and near IR regions. In specific, the intensity increase between 350 and 800 was gradually enhanced, however, in the NIR region, the reflectance was gradually decreased above 750°C. To observe the spectral evolution clearly, the average reflectance trend in UV-VIS and NIR regions were separately investigated as shown in Fig 4(D). As evidenced by Fig 4(D), the reflectance in UV-VIS and NIR region was consistently increased up to 750°C with the formation of overlaying NPs and dense isolated NPs. Between 750 and 850°C, the reflectance intensity in the UV-VIS region were almost similar (~ 19%), which can be induced by the enhancement from Pt nanostructure LSPR in the VIS region and at 900°C, the reflectance between 400 and 600 nm was further enhanced, showing a peak that matches the Pt dipolar position recorded between 500 and 550 nm. Lastly, the dipolar peak was still observed at 950°C as seen in Fig 4(B). Meanwhile, due to the slightly reduced average diameter as well as the nanostructure coverage, the overall reflectance intensity was decreased. Fig 4(E) displays the Raman spectral evolution of A_1g_ vibrational mode peaks of the samples [41] and the corresponding intensity, peak position and full width at half maximum (FWHM) are summarized in Fig 4(F), 4(G) and 4(H) accordingly. The specific values of Raman peak intensity, position and FWHM are summarized in S4 Table. The Raman spectral variation was also observed with the morphological evolution of Pt NSs. As seen in Fig 4(F), the decrease of peak intensity was witnessed between 600 and 750°C with the formation of isolated Pt NSs. As discussed above, owing to the surface plasmon effect, the light absorption can be enhanced which can result in the attenuation of peak intensity. Consequently, the intensity was weakened between 750 and 900°C, which matches with the UV-vis reflectance shown in Fig 4(D). At 950°C, as the reflectance was decreased, the Raman peak intensity was recovered as seen in Fig 4(E) and 4(F), which can be also induced by the slightly decreased nanostructure coverage. With respect to the peak position, the A_1g_ peaks appeared at ~ 417.6 cm^-1^ with the minor shift within 0.1 cm^-1^, suggesting the sapphire lattice strain induced by the Pt NSs fabrication stayed in a low level [42, 43]. In addition, the FWHMs were distributed stably between 8 and 8.5 cm^-1^.

**Fig 5:**
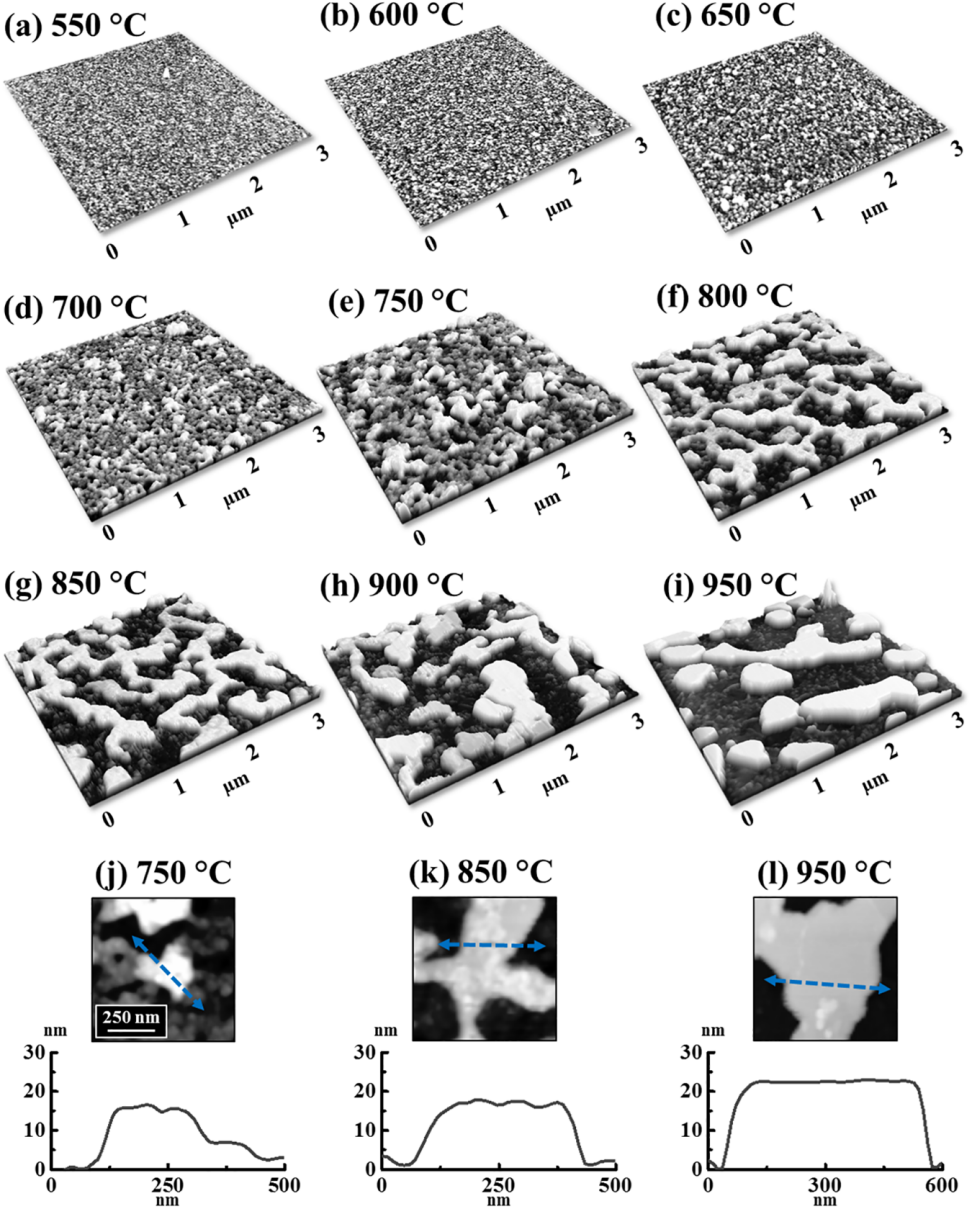
Annealing temperature effect on the surface morphology evolution of Pt nanostructures with 20 nm-thick Pt deposition annealed for constant 450 s at different temperature as labelled. (a)–(i) AFM side-views of 3 × 3 μm^2^. (j)–(l) Enlarged AFM top-views along with the corresponding line-profiles.

**Fig 6:**
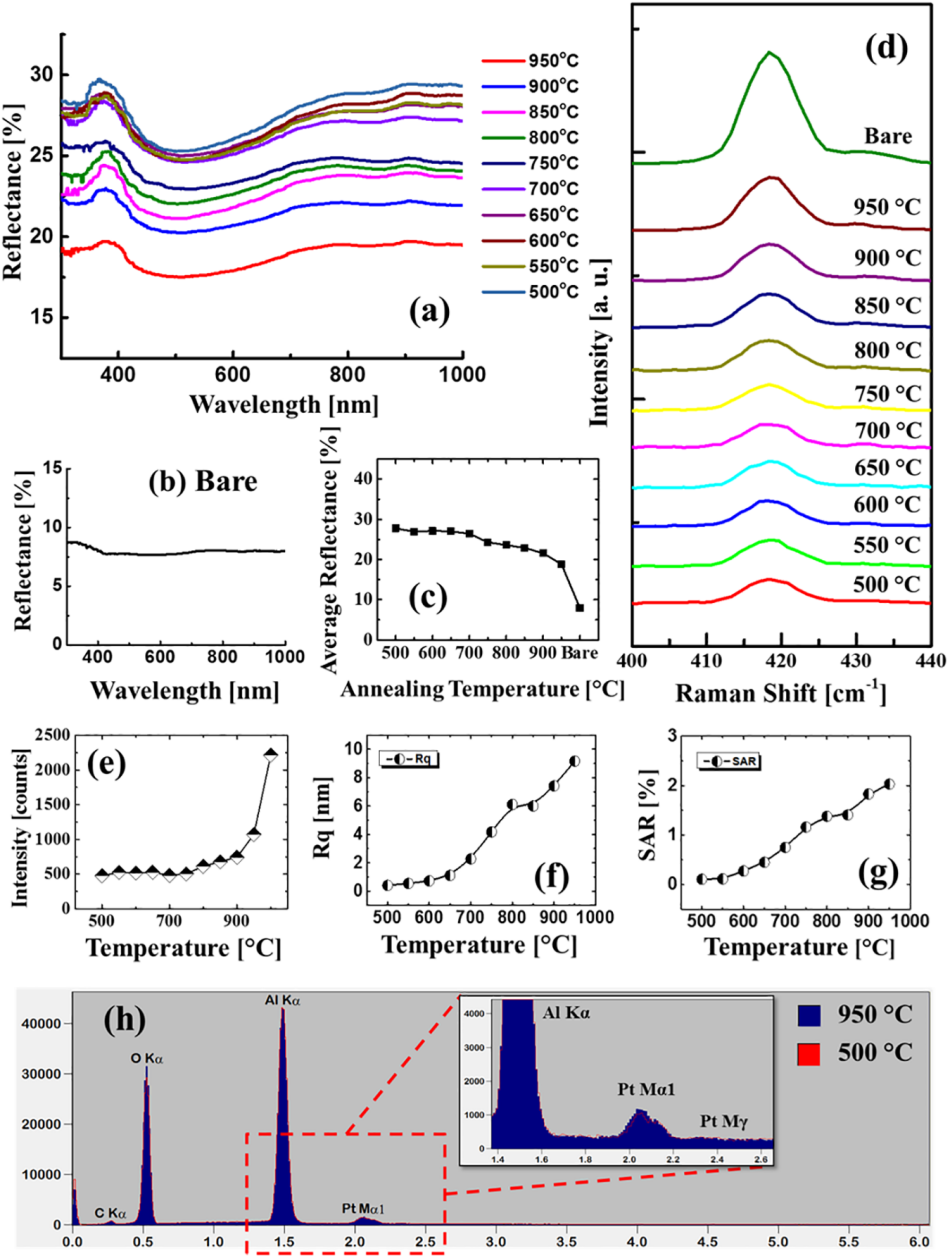
(a) Reflectance spectra of the Pt nanostructures on sapphire fabricated with the variation of annealing temperatures with the 20 nm Pt deposition. (b) Reflectance spectrum of bare sapphire. (c) Average reflectance plotted as a function of annealing temperature. (d) Corresponding Raman spectra of the A_1g_ peak. (e) Summary plot of A_1g_ peak intensities. Summary plots of (f) Rq and (g) SAR. (h) EDS elemental characterization of the samples at 950 and 500°C. (Inset) The enlarged energy range between 1.4 and 2.6 keV showing Pt peaks.

Fig 5 shows the evolution of self-assembled Pt NSs with the increased Pt thickness of 20 nm by the AT variation between 500 and 950°C. The corresponding AFM top-views of 3 × 3 μm^2^ and SEM images are provided in S5 and S6 Figs and the detailed analyses with the AFM top-, side-views and cross-sectional line profiles are presented in S7 Fig. As compared to the previous set, a similar evolution trend was observed at a relatively lower temperature range between 550 and 800°C, however, at increased temperature between 850 and 950°C the NSs showed a distinct evolution with the lateral growth and coalescence. For example, the nucleation of tiny voids and growth of overlaying Pt nanoclusters were observed between 550 and 650°C as clearly shown in Fig 5(A), 5(B) and 5(C) and the height of a typical nanocluster was ~ 7 nm. Then, between 700 and 800°C, along with the enhanced surface diffusion and void growth, the Pt layers gradually evolved into wiggly NSs at 800°C as seen in Fig 5(F). Finally, between 850 and 950°C, the wiggly NSs were evolved due to the significant void coalescence along with the agglomeration of Pt atoms in order to reduce the surface and interface energy [44]. As evidenced by the line-profiles shown in Fig 5(J) and 5(L), between 750 and 950°C, the lateral dimension of NSs was clearly expanded while the NS height was slightly increased from ~15 to 22 nm. Noticeably, as presented in Fig 5(L), the NSs were eventually formed with a flat top, which might be (111) plane due to the crystal structure of Pt and sapphire. As well known, the single-crystal sapphires possess the rhombohedral structure and on the *c*-plane, each Al (O) atom can be surrounded by six O (Al) atoms, forming a hexagonal arrangement [15]. Meanwhile, as the crystal structure of Pt is face centered cube (fcc), the growth of Pt NSs can follow [111] direction, along which the arrangement of Pt atoms can match the hexagonal arrangement of sapphire substrate. In addition, as the (111) plane contains the lowest surface energy among various crystal orientations [45] and thus the flat top could be preferentially grown (111) plane. In addition, the R_q_ and SAR are presented in Fig 6(F) and 6(G) and the corresponding values are summarized in S2 Table. The R_q_ and SAR were gradually increased along with the AT from 500 to 950°C suggesting the evolution of enlarged Pt NSs. Fig 6(H) displays the EDS spectra of 950 and 500°C samples and enlarged Pt Mα1 peaks are presented in the inset. The intensity of Pt Mα1 peaks was similar between 500 to 950°C, which confirms the equal amount of Pt in each sample despite of the indifferent morphology without any sublimation loss at high temperature. Furthermore, the NSs fabricated with 3 nm Pt deposition are presented in S9–S13 Figs, likewise, represented with the AFM top-, side- views, Rq, SAR and EDS spectra. The evolution of 3 nm set was distinct from 10 and 20 nm sets in that the formation of round-dome shaped Pt NPs were observed throughout the whole AT range between 500 and 950°C. Due to sufficient dewetting of low thickness film, the metal NSs tend to reduce the total surface energy by determining an equilibrium configuration for a certain volume based on the Gibbs-Wulf theorem and when the energy distribution is iosotropic [46], the round-dome shape can be obtained [15].

The reflectance and Raman spectral evolution of the corresponding Pt NSs with the 20 nm deposition are presented in Fig 6(A)–6(G). Similar to the 10 nm set, the fabrication of Pt NSs demonstrated enhanced reflectance as compared to the bare sapphire. Meanwhile, as clearly seen in Fig 6(A), the quadrupolar peak was similarly observed at ~ 380 nm and also the reflectance intensity was gradually decreased along with the increased temperature between 500 and 950°C, however, the dipolar peak was not observed throughout the temperature range, which sharply contrasts the result of 10 nm set. The difference of reflectance characteristics between 10 and 20 nm sets can be attributed to the distinct morphological evolution of Pt NSs. As the Pt films were still not developed into individual nanostructures even at 950°C, the resonance through dipole mode can be hindered. At the same time, as the surface coverage of Pt NSs was gradually reduced by the formation of voids and wiggly Pt nanoclusters along with the increased temperature as seen in Fig 5. Thus more area of sapphire substrate was exposed, which resulted in a decreasing reflectance. Fig 6(D) shows the Raman peak corresponding to the A_1g_ vibration mode and the corresponding intensity variation is summarized in Fig 6(E). Compared with 10 nm set, the Raman signal became much weaker with the higher surface coverage of Pt NSs likely due to the absorption of incident photon based on the dip observed in reflectance spectra. Also, the peak intensity was gradually increased between 500 and 950°C, which can be a correspondence with the gradually reduced coverage of Pt NSs.

**Fig 7:**
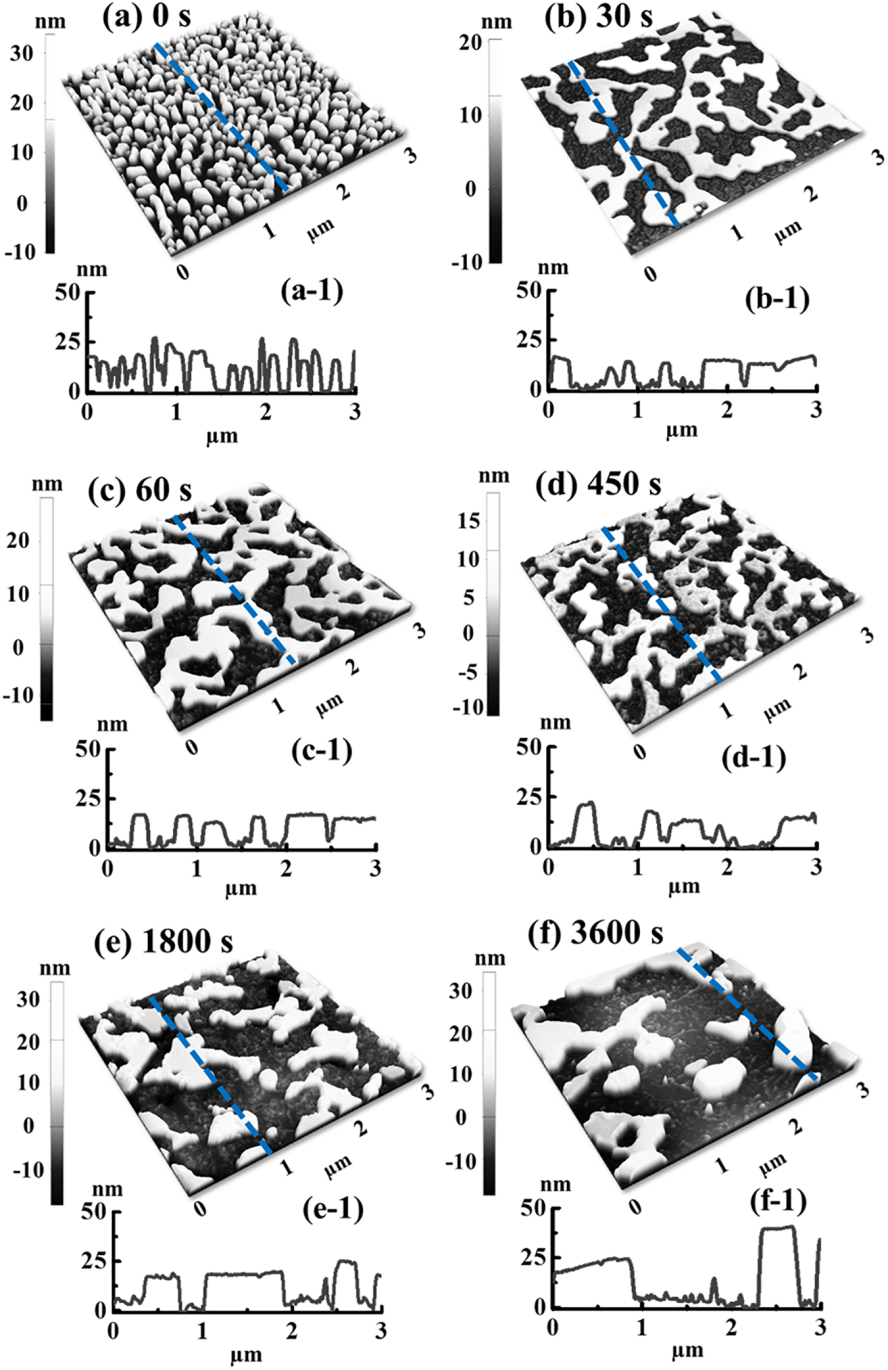
Dwelling time effect on the surface morphology evolution of Pt nanostructures with constant film thickness of 20 nm annealed at 800°C for 0 to 3600 s. (a)–(f) AFM side-views of 3 × 3 μm^2^. (a-1)–(f-1) Corresponding line-profiles.

**Fig 8:**
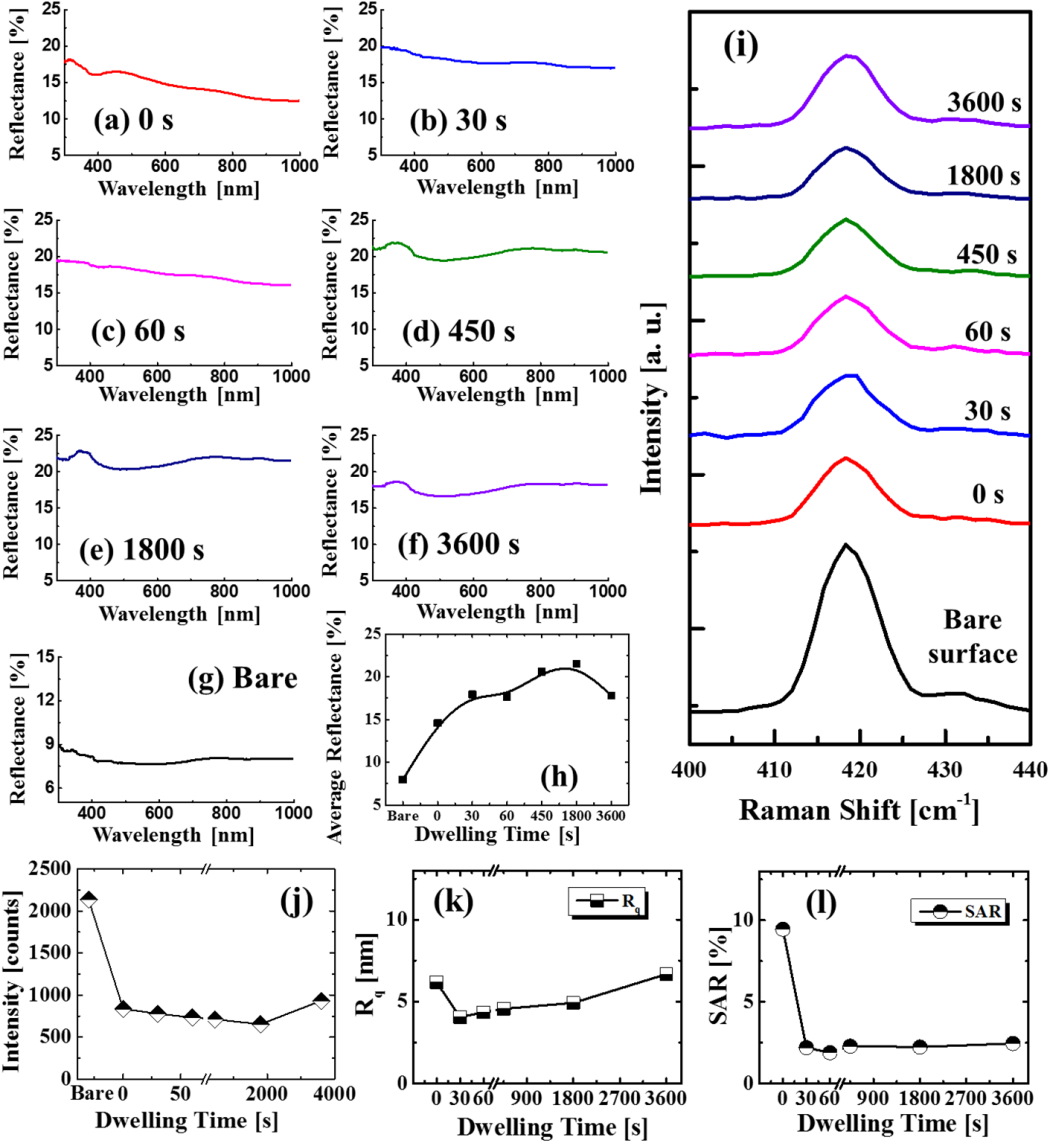
(a)–(f) Reflectance spectra Pt nanostructures on sapphire, fabricated with the 20 nm Pt thickness at 800°C for various dwelling time between 0 and 3600 s. (g) Reflectance spectrum of bare sapphire. (h) Average reflectance with respect to the dwelling time. (i) Raman spectra of A_1g_ peaks. Summary plots of (j) peak intensity (A_1g_), (k) Rq and (l) SAR.

Fig 7 shows the evolution of self-assembled Pt NSs based on the annealing duration control between 0 and 3600 s at 800°C with a fixed Pt deposition thickness of 20 nm. The corresponding AFM top-views of 5 × 5 μm^2^ are shown in S14 Fig. Firstly, the resulting Pt nanoparticles were merged in the first 30 s and then, gradually developed into wiggly nanostructures. Specifically, the wiggly Pt nanoclusters with packed density were first fabricated at 0 s as shown in Fig 7(A) which can be ascribed to the insufficient diffusion period. After increasing dwelling time, the Pt nanoclusters gradually merged into nanoclusters networks between 30 and 60 s as shown in Fig 7(B) and 7(C). Subsequently, the Pt nanocluster networks were broken into the isolated and widely spaced Pt nanoclusters adopting the wiggly configurations at 450 s with the energy perturbation such as Rayleigh instability [36]. Finally, with the extended dwelling duration of 1800 and 3600 s, the NSs gradually grew and merged into larger ones. According to the Ostwald ripening mechanism, the relatively larger NSs possessing lower surface energy can grow at the expense of absorbing smaller ones during the annealing in order to further decrease the overall surface energy [47, 48]. As a result, after 3600 s of annealing, the average height and lateral size were increased as seen in the Fig 7(F). The R_q_ and SAR of 20 nm time set are summarized in Fig 8(K) and 8(L) and the specific values are listed in S3 Table. The R_q_ and SAR were initially decreased between 0 and 30 s with the presence of Pt NP network as discussed and with further extended time, the R_q_ was gradually increased while SAR showed a slight increase due to the compensation between size and density. Additionally, the annealing duration effect was further investigated with the reduced film thickness of 15 nm at 800°C and the characterizations showed a similar trend to the 20 nm set as presented in S15–S18 Figs. Fig 8(A)–8(G) show the corresponding reflectance spectra of Pt NSs based on the annealing duration control. As seen in Fig 8(A), with the formation of dense NPs, the reflectance showed a relatively lower intensity which gradually decreased for longer wavelength, indicating the possible surface plasmon effect. With the formation of Pt NSs network at 30 s, the reflectance was drastically increased as seen in Fig 8(B) and then, as the NSs coverage was gradually reduced between 30 and 3600 s, a slight decrease was accordingly observed as shown by the summary plot in Fig 8(H). The corresponding Raman spectra are shown in Fig 8(I). As exhibited by the intensity evolution shown in Fig 8(J), the Raman signal was first decreased between 0 and 30 s and then increased between 30 and 3600 s, which can be correlated to the evolution of average surface coverage of Pt NSs. Likewise, no obvious shift of phonon modes was observed for the various samples.

## Conclusions

In summary, the evolution of various configurations, size and spacing of self-assembled Pt nanostructures (NSs) from the deposited Pt layers on *c*-plane sapphire (0001) were demonstrated by the control of annealing temperature, duration and Pt film thickness. By increasing annealing temperature, the Pt NSs were gradually formed from the continuous layers through the surface diffusion, agglomeration and surface-interface energy minimization. For the 10 nm initial thickness, at relatively lower temperature below 750°C, the nucleation and formation of overlaying Pt nanoclusters were observed. Whereas at increased temperature between 750 and 950°C, with the enhanced surface diffusion, the isolated Pt NSs were gradually fabricated according to the Volmer-Weber growth model. For the 20 nm set, the tiny voids and grains were resulted at low temperature, however, at increased temperature, the wiggly Pt NSs were formed according to the coalescence growth model. In the case of dwelling time variation, evolution of Pt NPs, networks-like and wiggly NSs were observed and discussed based on the surface energy minimization, Rayleigh instability as well as Ostwald ripening. Moreover, by the reflectance spectra analysis, reflectance enhancement and the evolution of wavelength dependent spectral response of Pt NSs were observed which can be induced by the surface plasmonic effect of Pt NSs. And, the evolution of Raman spectra was witnessed for all sets along with the morphological evolution. Finally, the process applied to the fabricated Pt NSs in this study can be employed in the practical applications by further exploiting their physical, optical and other related properties.

## Supporting information

S1 Fig (a) Raman spectra between 200 and 1000 cm^-1^ of bare sapphire excited by 532 nm laser at various power: 200 (rose red), 100 (blue), 50 (red) and 20 mW (black). The intensity of each excited peak was gradually enhanced with the increased laser power without a shift. According to the group theory, the optical modes of sapphire can be expressed as: Γ = 2*A*_1*g*_ + 2*A*_1*u*_ + 3*A*_2*g*_ + 2*A*_2*u*_ + 5*A*_*g*_ + 4*E*_*u*_.^1^ Five planar modes E_g_ peaks were detected at 378, 430, 451, 578, and 750 cm^-1^, respectively.^1,^ The peak appeared at 417 belongs to the A_1g_ mode.^2^ (b) Atomic force microscope (AFM) top-view of the bare sapphire (0001) (3 × 3 μm^2^). (b-1) Cross-sectional line-profile obtained by yellow line in (b). (b-2) Two-dimensional (2-D) Fourier filter transform (FFT) power spectrum.
(DOCX)

S2 Fig (a)—(d) AFM top-views of 3 × 3 μm^2^, showing the surface morphologies with the various deposition amount between 3 and 20 nm before annealing. (a-1)—(d-1) Line-profiles obtained from the yellow lines in (a)—(d). (Insets) 2-D FFT power spectra. (e) Root mean squared roughness (Rq). (f) Surface area ratio (SAR). The line profiles, Rq and SAR show the gradual enhancement of surface height, roughness and area increment with the deposition amount.
(DOCX)

S3 Fig (a)—(j) AFM side-views (3 × 3 μm^2^) of Pt nanostructures on sapphire fabricated between 500 and 950°C for constant 450 s and 10 nm Pt deposition.
(DOCX)

S4 Fig (a)—(j) Energy-dispersive x-ray spectroscopy (EDS) spectra of samples fabricated at various annealing temperatures between 500 and 950°C with the 10 nm Pt thickness (surface morphologies shown in S3 Fig). The peaks appeared at 2.051 keV are Pt Mα1.
(DOCX)

S5 Fig (a)—(j) AFM top-views of 3 × 3 μm^2^, showing the surface morphologies of Pt nanostructures fabricated with increased deposition amount (20 nm) by annealing at temperatures between 500 and 950°C for 450 s.
(DOCX)

S6 Fig (a)—(f) Scanning electron microscope (SEM) images (6.8 (x) × 5.2 (y) μm^2^) of the Pt nanostructures evolution between 700 and 950°C for 450 s with the 20 nm Pt thickness.
(DOCX)

S7 Fig Surface morphology evolution of Pt nanostructures along based on the annealing temperature control between 700 and 950°C for 450 s with the 20 nm Pt deposition. (a)—(f) AFM side-views of 1 × 1 μm^2^ and the corresponding top-views of 1 × 1 μm^2^ in (a-1)—(f-1). (a-2)—(f-2) Line-profiles obtained from the green lines in (a)—(f).
(DOCX)

S8 Fig EDS spectra of 20 nm Pt deposition set. The Pt nanostructures were fabricated by varying the annealing temperatures between 500 and 950°C.
(DOCX)

S9 Fig (a)—(j) AFM top-views of 3 × 3 μm^2^, showing the annealing temperature effect on the evolution of Pt NPs (3 nm-thickness Pt deposition). The fabrication was controlled by the variation of temperatures between 500 and 950°C for 450 s.
(DOCX)

S10 Fig Fabrication of self-assembled Pt NPs on sapphire (0001) from 3 nm thick Pt film annealed between 500 and 750°C for 450 s. (a)—(f) AFM side-views of 500 × 500 nm^2^. (a-1)—(f-1) AFM Top-views of 500 × 500 nm^2^. (a-2)—(f-2) Corresponding cross-sectional line-profiles.
(DOCX)

S11 Fig Self-assembled Pt NPs on sapphire (0001) fabricated at the higher temperature range between 800 and 950°C for 450 s with 3 nm total Pt thickness. (a)—(d) AFM side-views of 500 × 500 nm^2^. (a-1)—(d-1) Corresponding top-views of 500 × 500 nm^2^. (a-2)—(d-2) Cross-sectional line-profiles.
(DOCX)

S12 Fig Plots of the (a) Rq and (b) SAR. EDS spectra shows Pt Mα1 peaks (2.051 keV) of samples fabricated at various temperatures: (c) between 500 and 700°C and (d) between 750 and 950°C.
(DOCX)

S13 Fig EDS spectra of Pt nanostructures on sapphire, fabricated with the 3 nm Pt deposition between 500 and 950°C. (DOCX)

S14 Fig (a)—(f) AFM top-views of 5 × 5 μm^2^, showing the dwelling time effect on Pt nanostructure evolution (20 nm-thick Pt film). The fabrication was performed by the control of dwelling duration between 0 and 3600 s at a fixed temperature 800°C.
(DOCX)

S15 Fig Dwelling time effect on the evolution of Pt nanostructures with reduced Pt film thickness (15 nm) at constant temperature (800°C) by annealing between 0 and 3600 s. (a)—(e) AFM top-views of 3 × 3 μm^2^.
(DOCX)

S16 Fig (a)–(e) Corresponding AFM side-views of 3 × 3 μm^2^, showing the side-views of Pt nanostructures evolved by the annealing time variation between 0 and 3600 s at 800°C with 15 nm deposition thickness.
(DOCX)

S17 Fig (a)–(e) AFM side-views (1 × 1 μm^2^) of the Pt NPs formed by the annealing duration control from 0 to 3600 s at 800°C with 15 nm Pt deposition thickness. (a-1)–(e-1) Corresponding top-views. (a-2)–(e-2) Cross-sectional line-profiles.
(DOCX)

S18 Fig (a)–(f) Reflectance spectra of the Pt NPs on sapphire with variable annealing duration as labelled at 800°C and 15 nm initial Pt thickness. (g) Reflectance spectrum of bare sapphire. (h) Average reflectance with respect to the dwelling time. (i) Corresponding Raman spectra of A_1g_ peaks. Summary plots of (j) A_1g_ peak intensity, (k) Rq and (l) SAR.
(DOCX)

S1 Table Summary of the root mean squared roughness (Rq) and surface area ratio (SAR) with the various deposition amounts of Pt (pre-anneal). (DOCX)

S2 Table Summary of Rq and SAR of the Pt nanostructures on sapphire with 3, 10, and 20 nm Pt deposition (DA) after annealing between 500 and 950°C for 450 s. (DOCX)

S3 Table Summary of Rq and SAR of Pt nanostructures on sapphire with the dwelling time (DT) control between 0 and 3600 s having different initial thickness of Pt film. (DOCX)

S4 Table Summary of Raman intensity of Pt nanostructures on sapphire for the annealing temperatures (AT) control sets (10 and 20 nm). Samples were fabricated with a variation of annealing temperatures (ATs) between 500 and 950°C.
(DOCX)

S5 Table Summary of Raman intensity of the Pt nanostructures on sapphire for the dwelling time (DT) control sets (15 and 20 nm). Samples were fabricated by the control of (DT) between 0 and 3600 s at a fixed annealing temperature (AT) of 800°C.
(DOCX)
