# Supplementary material for: Effects of annealing temperature and duration on the morphological and optical evolution of self-assembled Pt nanostructures on c-plane sapphire
Source: PLoS One. 2017 May 4;12(5):e0177048. doi: 10.1371/journal.pone.0177048 (PMC5417639; doi:10.1371/journal.pone.0177048)
Supplement: S1 Fig — (a) Raman spectra between 200 and 1000 cm-1 of bare sapphire excited by 532 nm laser at various power: 200 (rose red), 100 (blue), 50 (red) and 20 mW (black). The intensity of each excited peak was gradually enhanced with the increased laser power without a shift. According to the group theory, the optical modes of sapphire can be expressed as: Γ = 2A1g + 2A1u + 3A2g + 2A2u + 5Ag + 4Eu.1 Five planar modes Eg peaks were detected at 378, 430, 451, 578, and 750 cm-1, respectively.1, The peak appeared at 417 belongs to the A1g mode.2 (b) Atomic force microscope (AFM) top-view of the bare sapphire (0001) (3 × 3 μm2). (b-1) Cross-sectional line-profile obtained by yellow line in (b). (b-2) Two-dimensional (2-D) Fourier filter transform (FFT) power spectrum. (DOCX) [file pone.0177048.s001.docx]

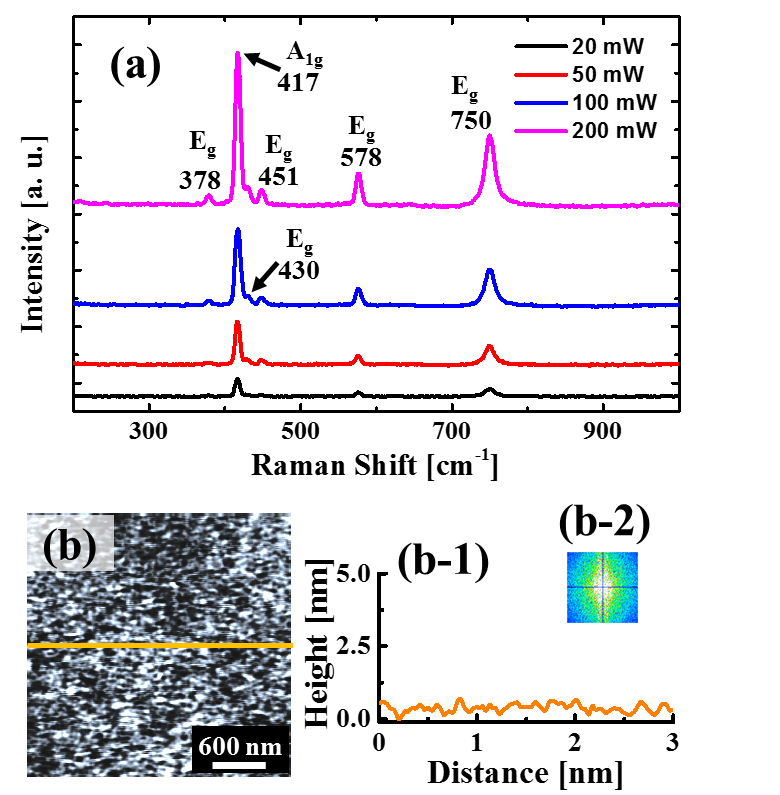


**S1 Fig.** (a) Raman spectra between 200 and 1000 cm^-1^ of bare sapphire excited by 532 nm laser at various power: 200 (rose red), 100 (blue), 50 (red) and 20 mW (black). The intensity of each excited peak was gradually enhanced with the increased laser power without a shift. According to the group theory, the optical modes of sapphire can be expressed as: $\Gamma={2A}_{1g}+{2A}_{1u}+{3A}_{2g}+{2A}_{2u}+{5A}_{g}+{4E}_{u}$ .^1^ Five planar modes E_g_ peaks were detected at 378, 430, 451, 578, and 750 cm^-1^, respectively.^1,^ The peak appeared at 417 belongs to the A_1g_ mode.^2^ (b) Atomic force microscope (AFM) top-view of the bare sapphire (0001) (3 × 3 µm^2^). (b-1) Cross-sectional line-profile obtained by yellow line in (b). (b-2) Two-dimensional (2-D) Fourier filter transform (FFT) power spectrum.

**Reference**

1. W. Zhu, and G. Pezzotti, J. Appl. Phys. **109**, 073502 (2011).

2. G. Pezzotti, and W. Zhu, Phys. Chem. Chem. Phys. **17**, 2608 (2015).
