# Supplementary material for: Effects of annealing temperature and duration on the morphological and optical evolution of self-assembled Pt nanostructures on c-plane sapphire
Source: PLoS One. 2017 May 4;12(5):e0177048. doi: 10.1371/journal.pone.0177048 (PMC5417639; doi:10.1371/journal.pone.0177048)
Supplement: S2 Fig — (a)—(d) AFM top-views of 3 × 3 μm2, showing the surface morphologies with the various deposition amount between 3 and 20 nm before annealing. (a-1)—(d-1) Line-profiles obtained from the yellow lines in (a)—(d). (Insets) 2-D FFT power spectra. (e) Root mean squared roughness (Rq). (f) Surface area ratio (SAR). The line profiles, Rq and SAR show the gradual enhancement of surface height, roughness and area increment with the deposition amount. (DOCX) [file pone.0177048.s002.docx]

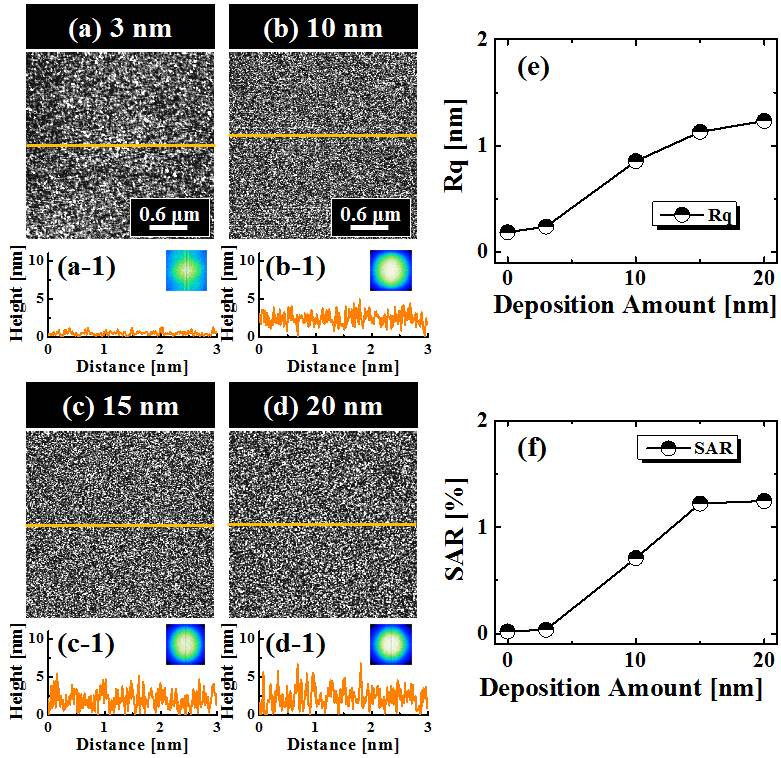


**S2 Fig.** (a) - (d) AFM top-views of 3 × 3 µm^2^, showing the surface morphologies with the various deposition amount between 3 and 20 nm before annealing. (a-1) - (d-1) Line-profiles obtained from the yellow lines in (a) - (d). (Insets) 2-D FFT power spectra. (e) Root mean squared roughness (Rq). (f) Surface area ratio (SAR). The line profiles, Rq and SAR show the gradual enhancement of surface height, roughness and area increment with the deposition amount.
