# Supplementary material for: Effects of annealing temperature and duration on the morphological and optical evolution of self-assembled Pt nanostructures on c-plane sapphire
Source: PLoS One. 2017 May 4;12(5):e0177048. doi: 10.1371/journal.pone.0177048 (PMC5417639; doi:10.1371/journal.pone.0177048)
Supplement: S3 Fig — (a)—(j) AFM side-views (3 × 3 μm2) of Pt nanostructures on sapphire fabricated between 500 and 950°C for constant 450 s and 10 nm Pt deposition. (DOCX) [file pone.0177048.s003.docx]

**
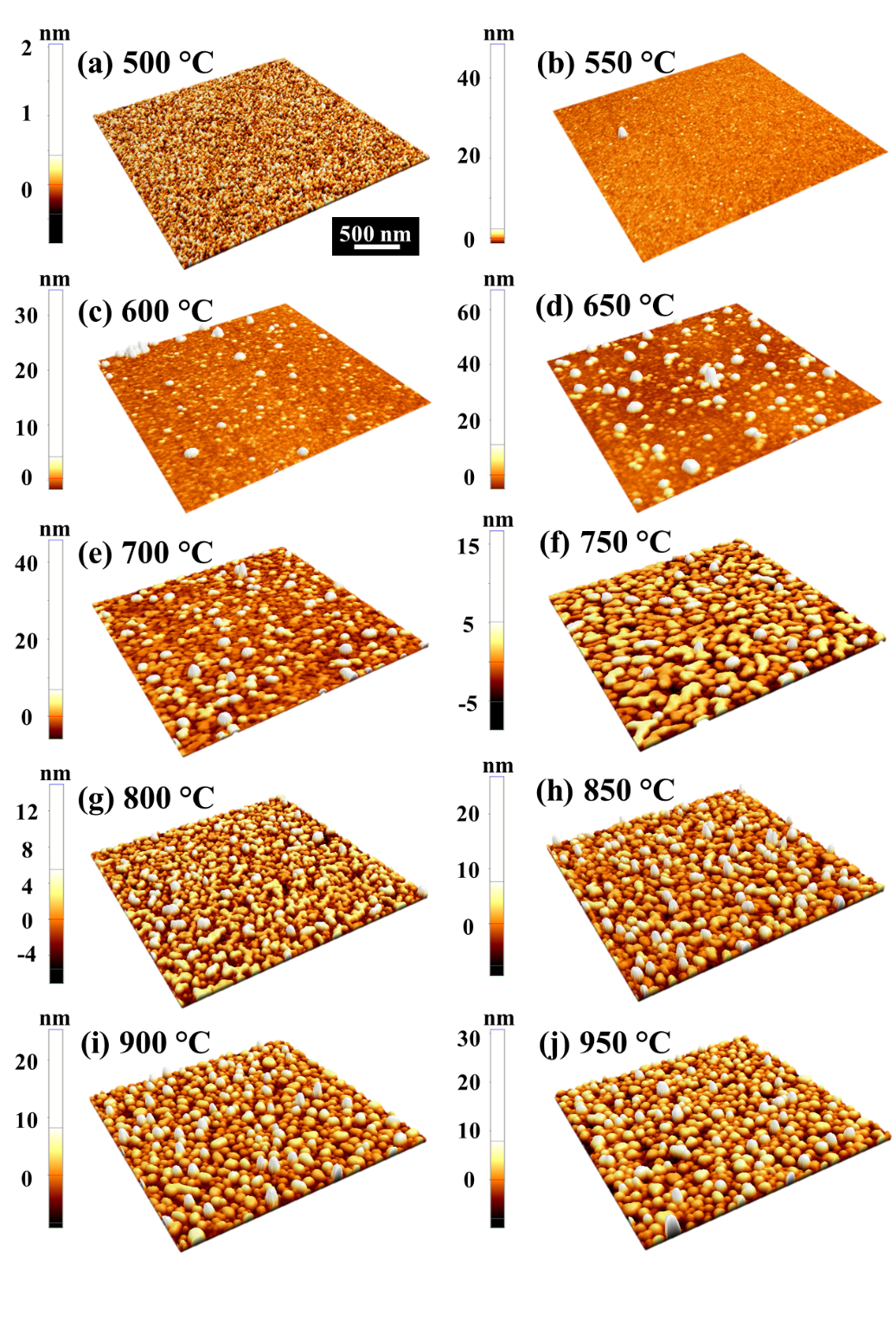
**

**S3 Fig.** (a) - (j) AFM side-views (3 × 3 µm^2^) of Pt nanostructures on sapphire fabricated between 500 and 950 ˚C for constant 450 s and 10 nm Pt deposition.
