# Supplementary material for: Effects of annealing temperature and duration on the morphological and optical evolution of self-assembled Pt nanostructures on c-plane sapphire
Source: PLoS One. 2017 May 4;12(5):e0177048. doi: 10.1371/journal.pone.0177048 (PMC5417639; doi:10.1371/journal.pone.0177048)
Supplement: S4 Fig — (a)—(j) Energy-dispersive x-ray spectroscopy (EDS) spectra of samples fabricated at various annealing temperatures between 500 and 950°C with the 10 nm Pt thickness (surface morphologies shown in S3 Fig). The peaks appeared at 2.051 keV are Pt Mα1. (DOCX) [file pone.0177048.s004.docx]

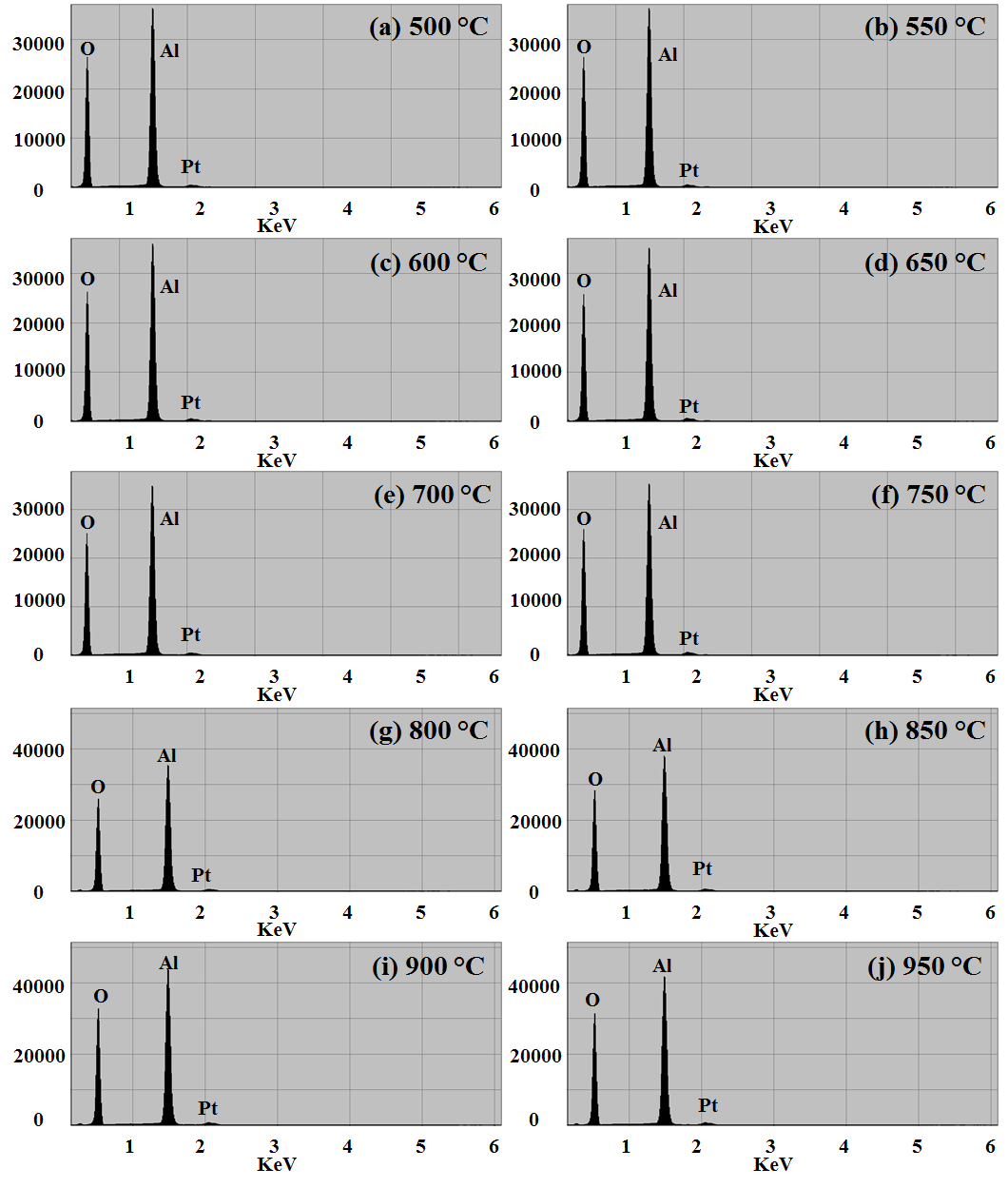


**S4 Fig.** (a) - (j) Energy-dispersive x-ray spectroscopy (EDS) spectra of samples fabricated at various annealing temperatures between 500 and 950 °C with the 10 nm Pt thickness (surface morphologies shown in Fig. S3). The peaks appeared at 2.051 keV are Pt Mα1.
