# Supplementary material for: Effects of annealing temperature and duration on the morphological and optical evolution of self-assembled Pt nanostructures on c-plane sapphire
Source: PLoS One. 2017 May 4;12(5):e0177048. doi: 10.1371/journal.pone.0177048 (PMC5417639; doi:10.1371/journal.pone.0177048)
Supplement: S5 Fig — (a)—(j) AFM top-views of 3 × 3 μm2, showing the surface morphologies of Pt nanostructures fabricated with increased deposition amount (20 nm) by annealing at temperatures between 500 and 950°C for 450 s. (DOCX) [file pone.0177048.s005.docx]

**
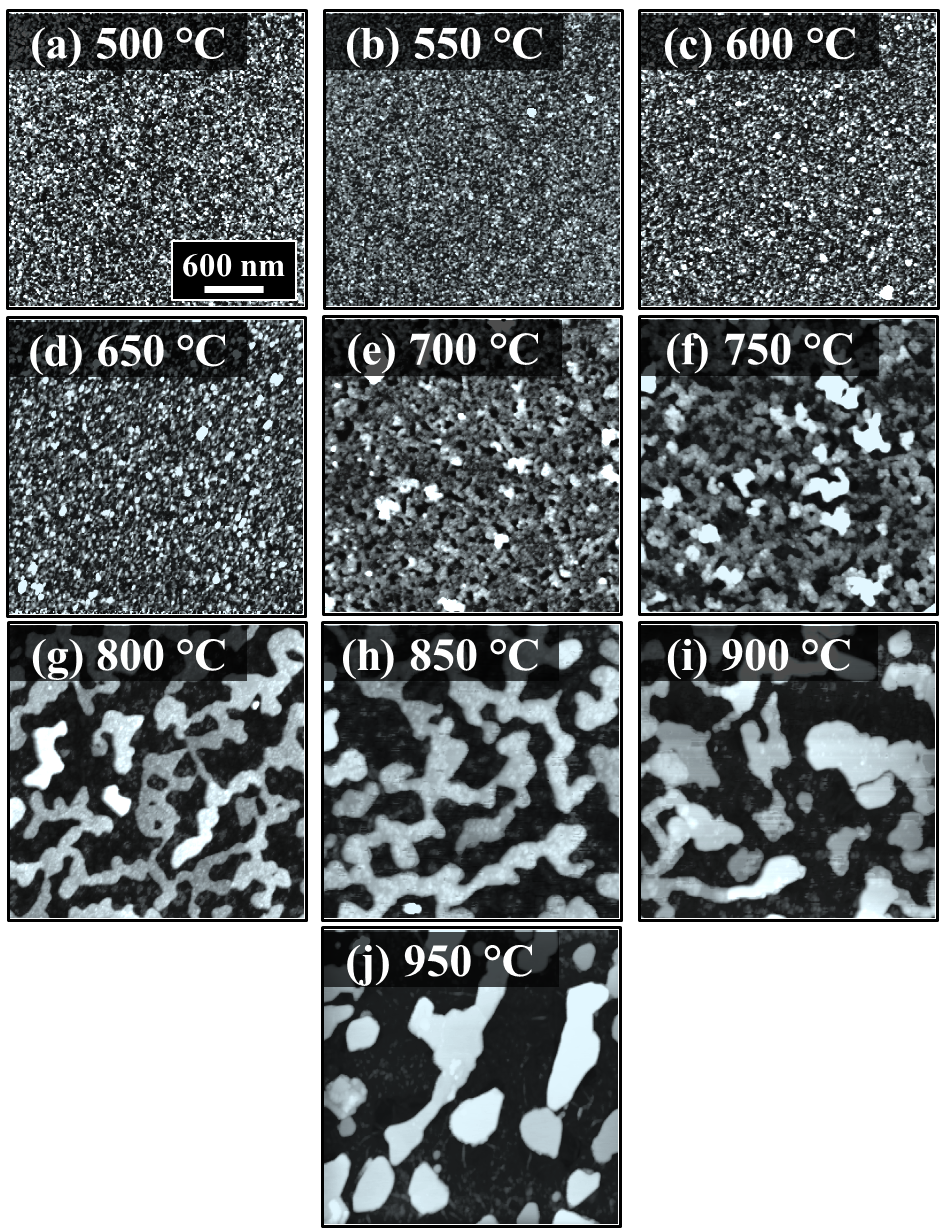
**

**S5 Fig.** (a) - (j) AFM top-views of 3 × 3 µm^2^, showing the surface morphologies of Pt nanostructures fabricated with increased deposition amount (20 nm) by annealing at temperatures between 500 and 950 ˚C for 450 s.
