# Supplementary material for: Effects of annealing temperature and duration on the morphological and optical evolution of self-assembled Pt nanostructures on c-plane sapphire
Source: PLoS One. 2017 May 4;12(5):e0177048. doi: 10.1371/journal.pone.0177048 (PMC5417639; doi:10.1371/journal.pone.0177048)
Supplement: S6 Fig — (a)—(f) Scanning electron microscope (SEM) images (6.8 (x) × 5.2 (y) μm2) of the Pt nanostructures evolution between 700 and 950°C for 450 s with the 20 nm Pt thickness. (DOCX) [file pone.0177048.s006.docx]

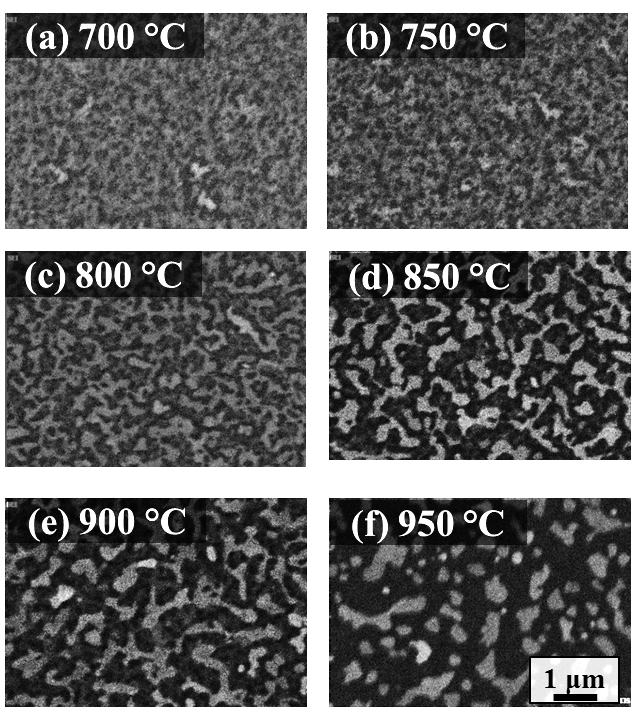


**S6 Fig.** (a) - (f) Scanning electron microscope (SEM) images (6.8 (x) × 5.2 (y) µm^2^) of the Pt nanostructures evolution between 700 and 950 ˚C for 450 s with the 20 nm Pt thickness
