# Supplementary material for: Effects of annealing temperature and duration on the morphological and optical evolution of self-assembled Pt nanostructures on c-plane sapphire
Source: PLoS One. 2017 May 4;12(5):e0177048. doi: 10.1371/journal.pone.0177048 (PMC5417639; doi:10.1371/journal.pone.0177048)
Supplement: S7 Fig — (a)—(f) AFM side-views of 1 × 1 μm2 and the corresponding top-views of 1 × 1 μm2 in (a-1)—(f-1). (a-2)—(f-2) Line-profiles obtained from the green lines in (a)—(f). (DOCX) [file pone.0177048.s007.docx]

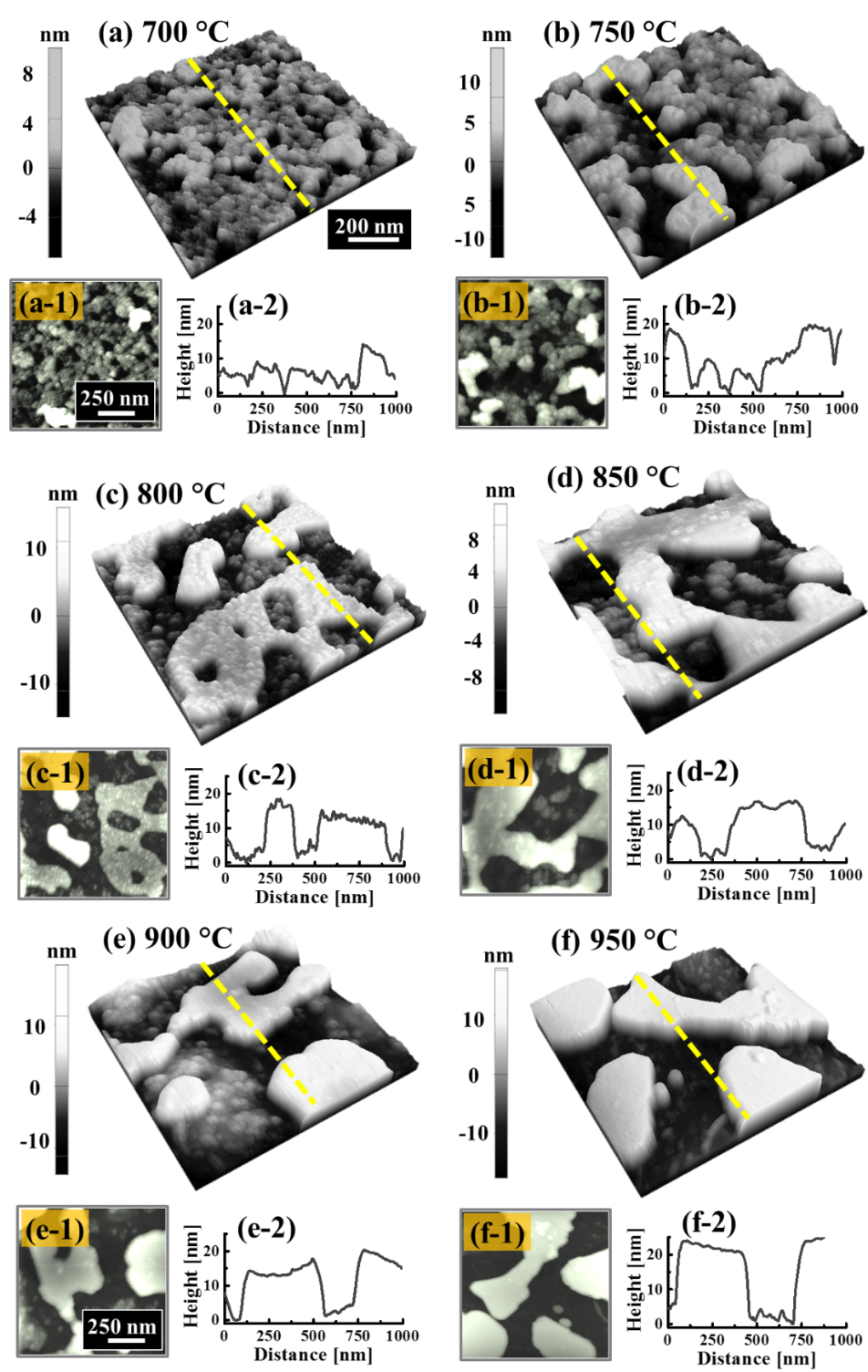


**S7 Fig.** Surface morphology evolution of Pt nanostructures along based on the annealing temperature control between 700 and 950 ˚C for 450 s with the 20 nm Pt deposition. (a) - (f) AFM side-views of 1 × 1 µm^2^ and the corresponding top-views of 1 × 1 µm^2^ in (a-1) - (f-1). (a-2) - (f-2) Line-profiles obtained from the green lines in (a) - (f).
