# Supplementary material for: Effects of annealing temperature and duration on the morphological and optical evolution of self-assembled Pt nanostructures on c-plane sapphire
Source: PLoS One. 2017 May 4;12(5):e0177048. doi: 10.1371/journal.pone.0177048 (PMC5417639; doi:10.1371/journal.pone.0177048)
Supplement: S8 Fig — The Pt nanostructures were fabricated by varying the annealing temperatures between 500 and 950°C. (DOCX) [file pone.0177048.s008.docx]

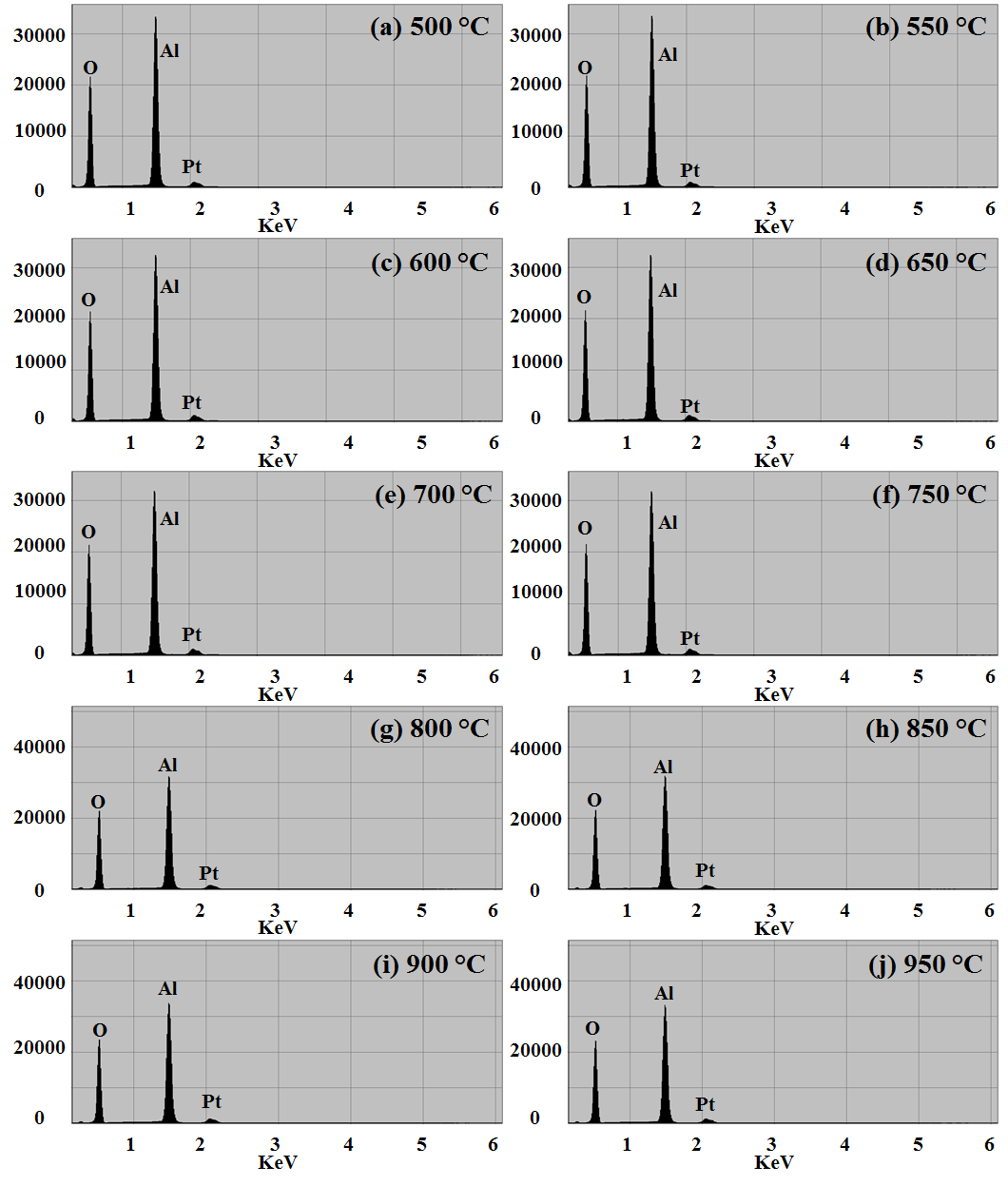


**S8 Fig.** EDS spectra of 20 nm Pt deposition set. The Pt nanostructures were fabricated by varying the annealing temperatures between 500 and 950 °C.
