# Supplementary material for: Effects of annealing temperature and duration on the morphological and optical evolution of self-assembled Pt nanostructures on c-plane sapphire
Source: PLoS One. 2017 May 4;12(5):e0177048. doi: 10.1371/journal.pone.0177048 (PMC5417639; doi:10.1371/journal.pone.0177048)
Supplement: S9 Fig — (a)—(j) AFM top-views of 3 × 3 μm2, showing the annealing temperature effect on the evolution of Pt NPs (3 nm-thickness Pt deposition). The fabrication was controlled by the variation of temperatures between 500 and 950°C for 450 s. (DOCX) [file pone.0177048.s009.docx]

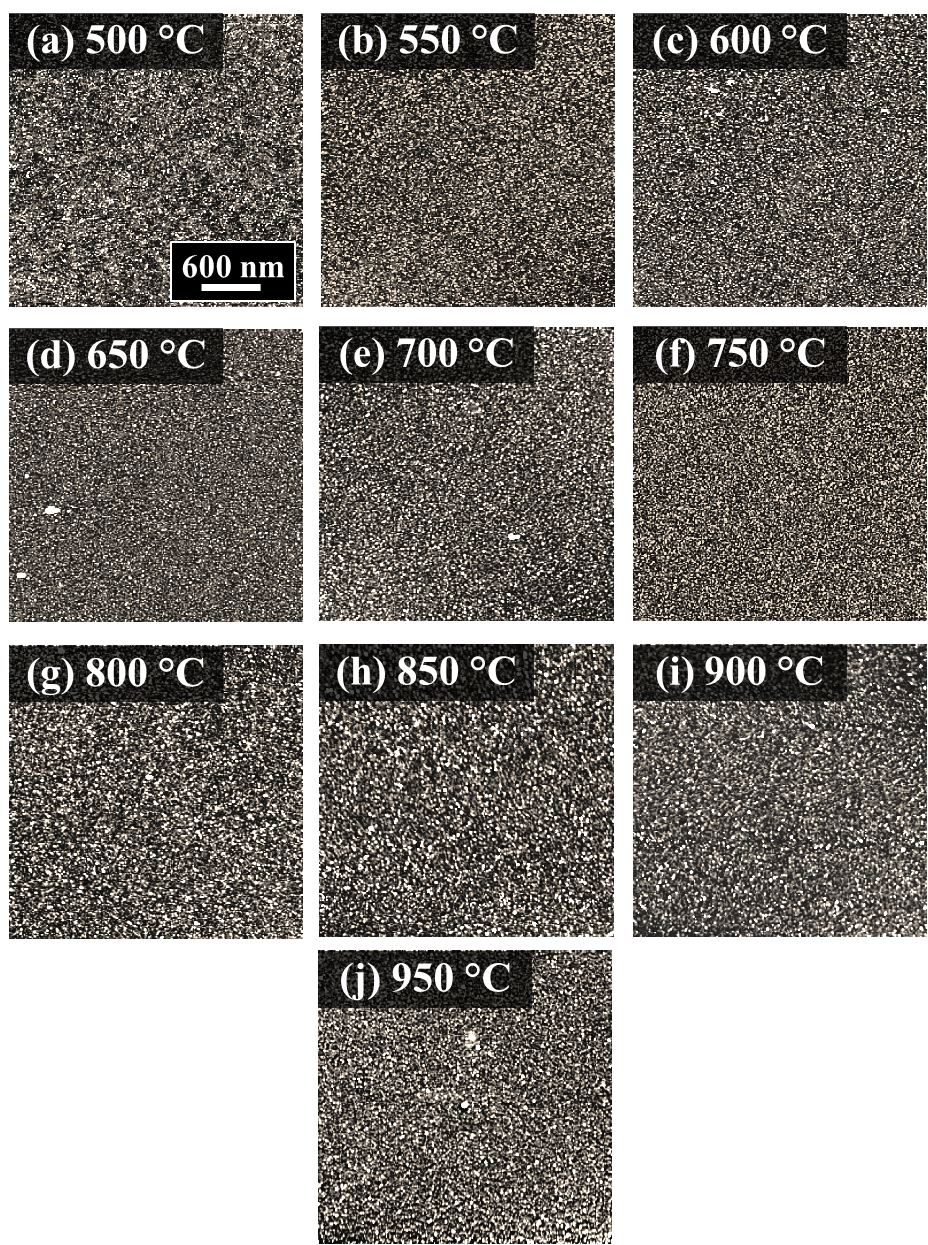


**S9 Fig.** (a) - (j) AFM top-views of 3 × 3 µm^2^, showing the annealing temperature effect on the evolution of Pt NPs (3 nm-thickness Pt deposition). The fabrication was controlled by the variation of temperatures between 500 and 950 ˚C for 450 s.
