# Supplementary material for: Effects of annealing temperature and duration on the morphological and optical evolution of self-assembled Pt nanostructures on c-plane sapphire
Source: PLoS One. 2017 May 4;12(5):e0177048. doi: 10.1371/journal.pone.0177048 (PMC5417639; doi:10.1371/journal.pone.0177048)
Supplement: S10 Fig — (a)—(f) AFM side-views of 500 × 500 nm2. (a-1)—(f-1) AFM Top-views of 500 × 500 nm2. (a-2)—(f-2) Corresponding cross-sectional line-profiles. (DOCX) [file pone.0177048.s010.docx]

**
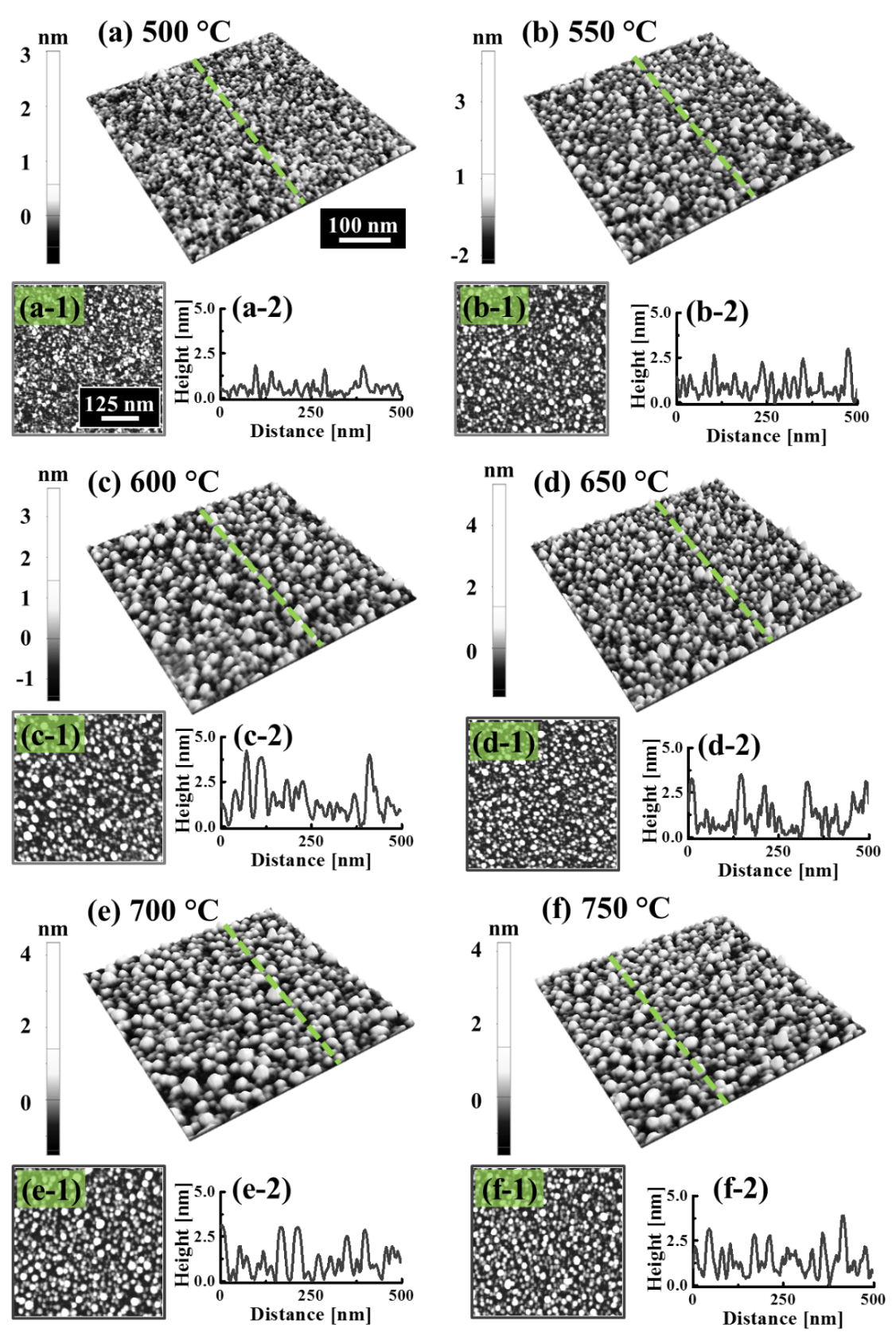
**

**S10 Fig.** Fabrication of self-assembled Pt NPs on sapphire (0001) from 3 nm thick Pt film annealed between 500 and 750 ˚C for 450 s. (a) - (f) AFM side-views of 500 × 500 nm^2^. (a-1) - (f-1) AFM Top-views of 500 × 500 nm^2^. (a-2) - (f-2) Corresponding cross-sectional line-profiles.
