# Supplementary material for: Effects of annealing temperature and duration on the morphological and optical evolution of self-assembled Pt nanostructures on c-plane sapphire
Source: PLoS One. 2017 May 4;12(5):e0177048. doi: 10.1371/journal.pone.0177048 (PMC5417639; doi:10.1371/journal.pone.0177048)
Supplement: S11 Fig — (a)—(d) AFM side-views of 500 × 500 nm2. (a-1)—(d-1) Corresponding top-views of 500 × 500 nm2. (a-2)—(d-2) Cross-sectional line-profiles. (DOCX) [file pone.0177048.s011.docx]

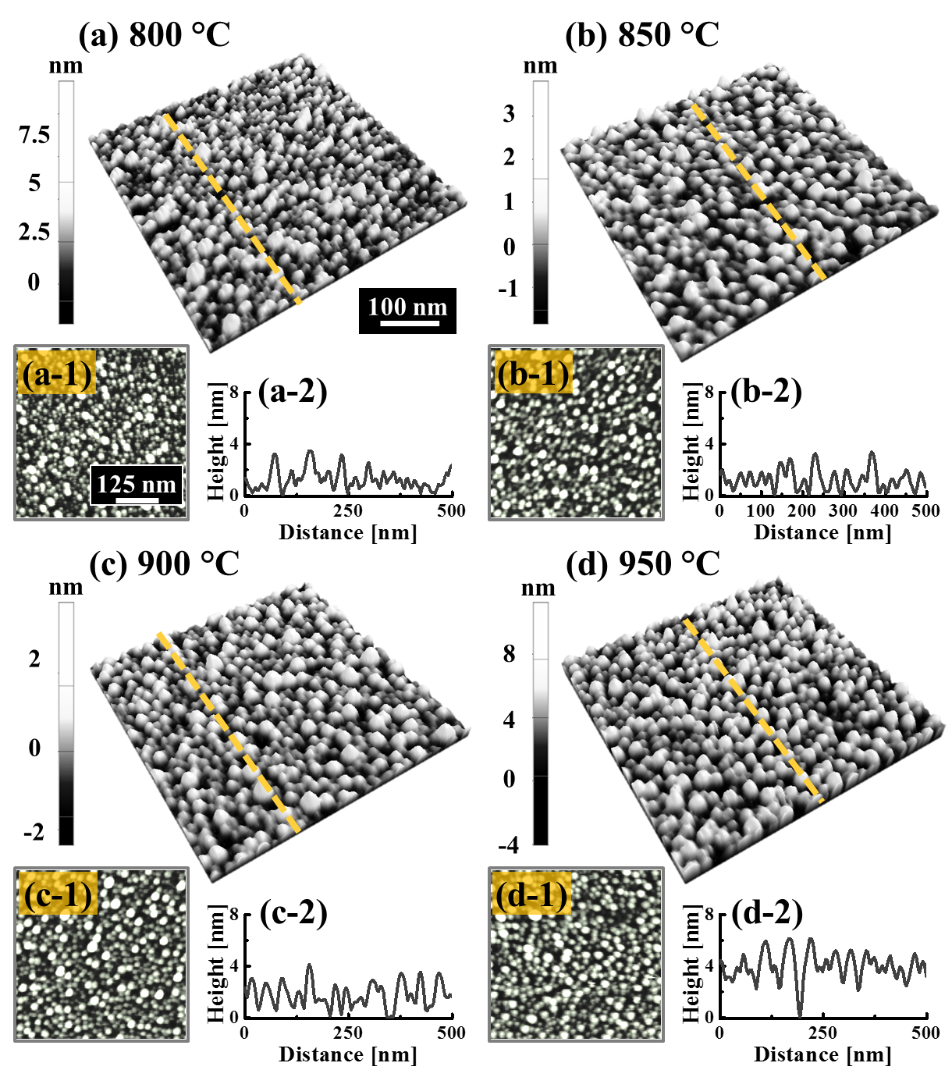


**S11 Fig.** Self-assembled Pt NPs on sapphire (0001) fabricated at the higher temperature range between 800 and 950 ˚C for 450 s with 3 nm total Pt thickness. (a) - (d) AFM side-views of 500 × 500 nm^2^. (a-1) - (d-1) Corresponding top-views of 500 × 500 nm^2^. (a-2) - (d-2) Cross-sectional line-profiles.
