# Supplementary material for: Effects of annealing temperature and duration on the morphological and optical evolution of self-assembled Pt nanostructures on c-plane sapphire
Source: PLoS One. 2017 May 4;12(5):e0177048. doi: 10.1371/journal.pone.0177048 (PMC5417639; doi:10.1371/journal.pone.0177048)
Supplement: S12 Fig — Plots of the (a) Rq and (b) SAR. EDS spectra shows Pt Mα1 peaks (2.051 keV) of samples fabricated at various temperatures: (c) between 500 and 700°C and (d) between 750 and 950°C. (DOCX) [file pone.0177048.s012.docx]

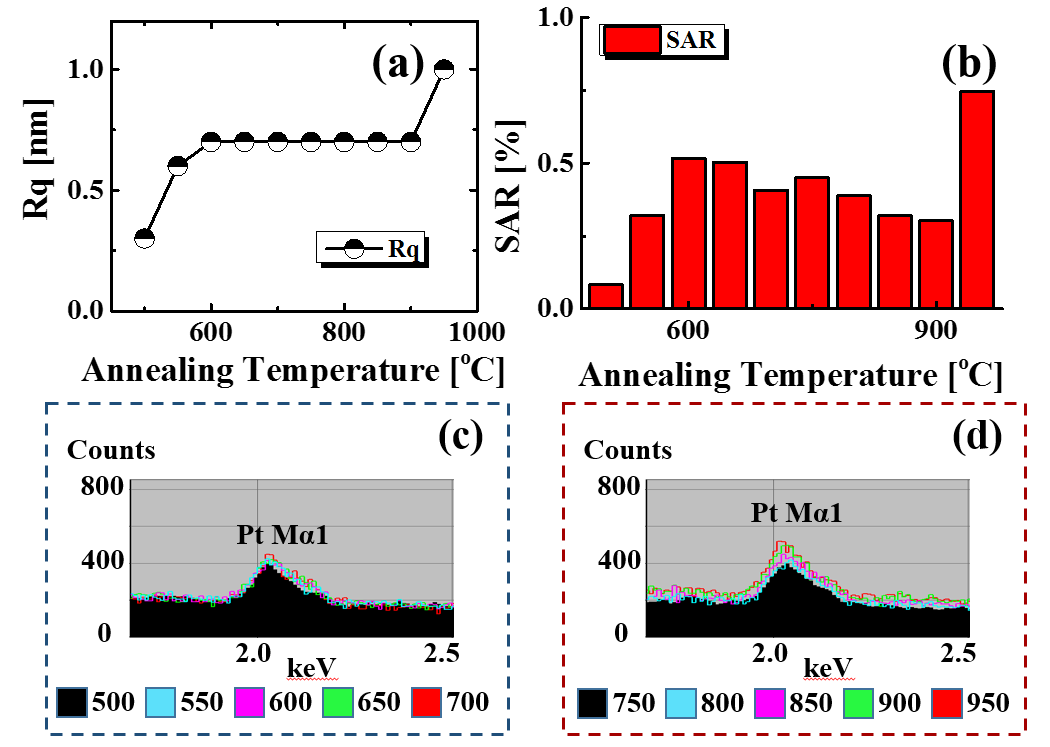


**S12 Fig.** Plots of the (a) Rq and (b) SAR. EDS spectra shows Pt Mα1 peaks (2.051 keV) of samples fabricated at various temperatures: (c) between 500 and 700 °C and (d) between 750 and 950 °C.
