# Supplementary material for: Effects of annealing temperature and duration on the morphological and optical evolution of self-assembled Pt nanostructures on c-plane sapphire
Source: PLoS One. 2017 May 4;12(5):e0177048. doi: 10.1371/journal.pone.0177048 (PMC5417639; doi:10.1371/journal.pone.0177048)
Supplement: S14 Fig — (a)—(f) AFM top-views of 5 × 5 μm2, showing the dwelling time effect on Pt nanostructure evolution (20 nm-thick Pt film). The fabrication was performed by the control of dwelling duration between 0 and 3600 s at a fixed temperature 800°C. (DOCX) [file pone.0177048.s014.docx]

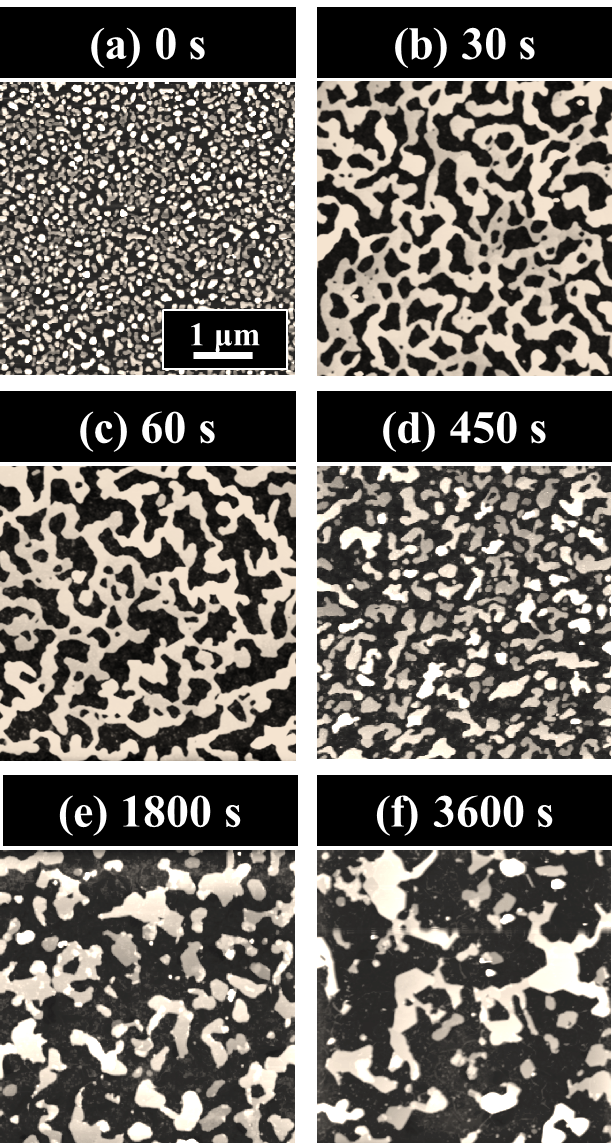


**S14 Fig.** (a) - (f) AFM top-views of 5 × 5 µm^2^, showing the dwelling time effect on Pt nanostructure evolution (20 nm-thick Pt film). The fabrication was performed by the control of dwelling duration between 0 and 3600 s at a fixed temperature 800 ˚C.
