# Supplementary material for: Effects of annealing temperature and duration on the morphological and optical evolution of self-assembled Pt nanostructures on c-plane sapphire
Source: PLoS One. 2017 May 4;12(5):e0177048. doi: 10.1371/journal.pone.0177048 (PMC5417639; doi:10.1371/journal.pone.0177048)
Supplement: S15 Fig — (a)—(e) AFM top-views of 3 × 3 μm2. (DOCX) [file pone.0177048.s015.docx]

**
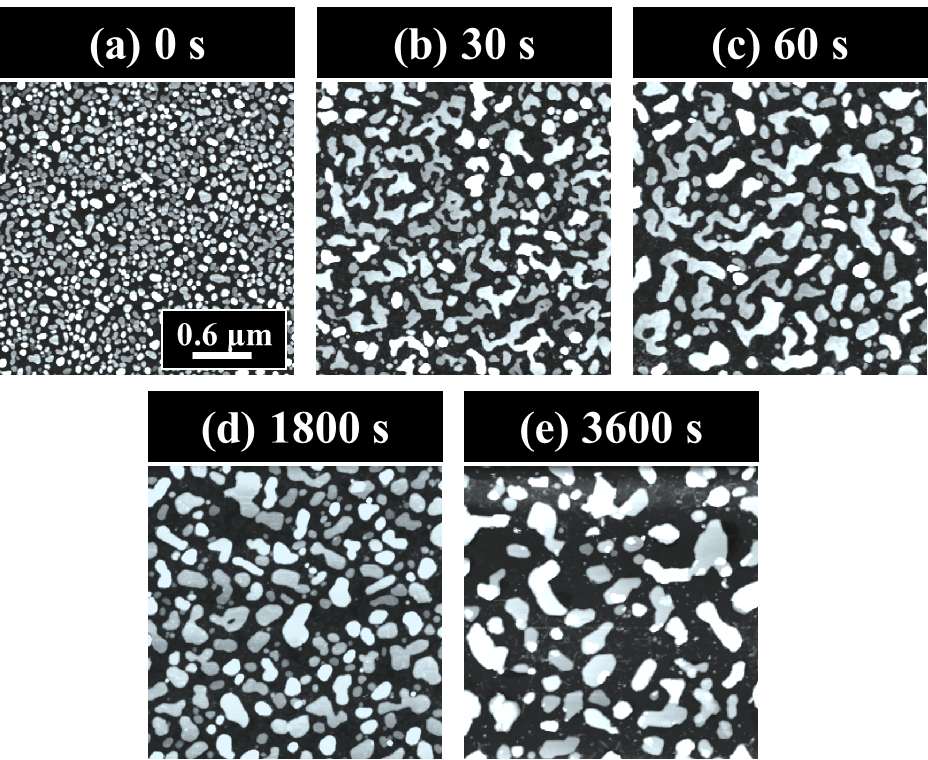
**

**S15 Fig.** Dwelling time effect on the evolution of Pt nanostructures with reduced Pt film thickness (15 nm) at constant temperature (800 °C) by annealing between 0 and 3600 s. (a) - (e) AFM top-views of 3 × 3 µm^2^.
