# Supplementary material for: Effects of annealing temperature and duration on the morphological and optical evolution of self-assembled Pt nanostructures on c-plane sapphire
Source: PLoS One. 2017 May 4;12(5):e0177048. doi: 10.1371/journal.pone.0177048 (PMC5417639; doi:10.1371/journal.pone.0177048)
Supplement: S16 Fig — (a)–(e) Corresponding AFM side-views of 3 × 3 μm2, showing the side-views of Pt nanostructures evolved by the annealing time variation between 0 and 3600 s at 800°C with 15 nm deposition thickness. (DOCX) [file pone.0177048.s016.docx]

**
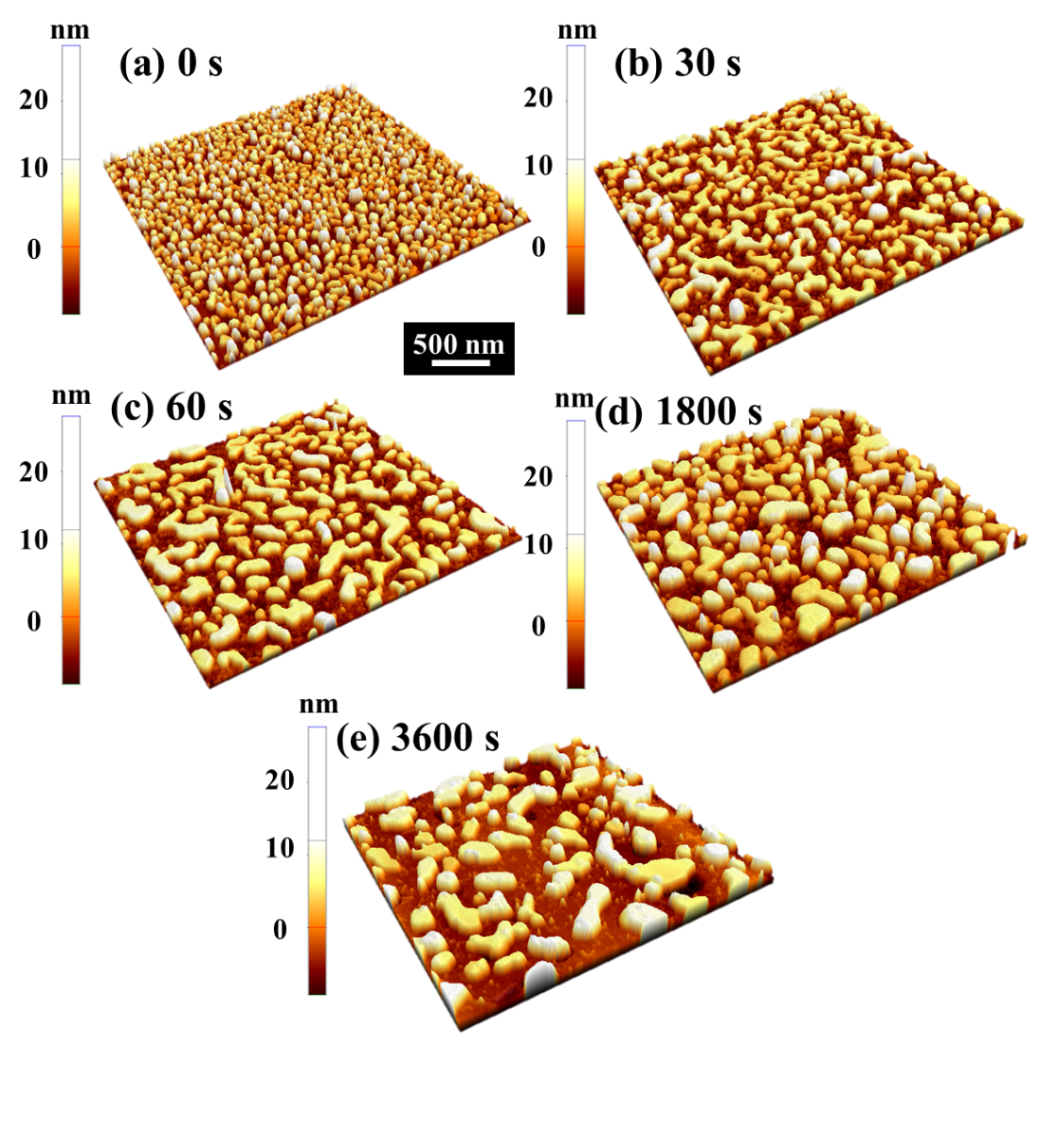
**

**S16 Fig.** (a) – (e) Corresponding AFM side-views of 3 × 3 µm^2^, showing the side-views of Pt nanostructures evolved by the annealing time variation between 0 and 3600 s at 800 ˚C with 15 nm deposition thickness.
