# Supplementary material for: Effects of annealing temperature and duration on the morphological and optical evolution of self-assembled Pt nanostructures on c-plane sapphire
Source: PLoS One. 2017 May 4;12(5):e0177048. doi: 10.1371/journal.pone.0177048 (PMC5417639; doi:10.1371/journal.pone.0177048)
Supplement: S17 Fig — (a)–(e) AFM side-views (1 × 1 μm2) of the Pt NPs formed by the annealing duration control from 0 to 3600 s at 800°C with 15 nm Pt deposition thickness. (a-1)–(e-1) Corresponding top-views. (a-2)–(e-2) Cross-sectional line-profiles. (DOCX) [file pone.0177048.s017.docx]

**
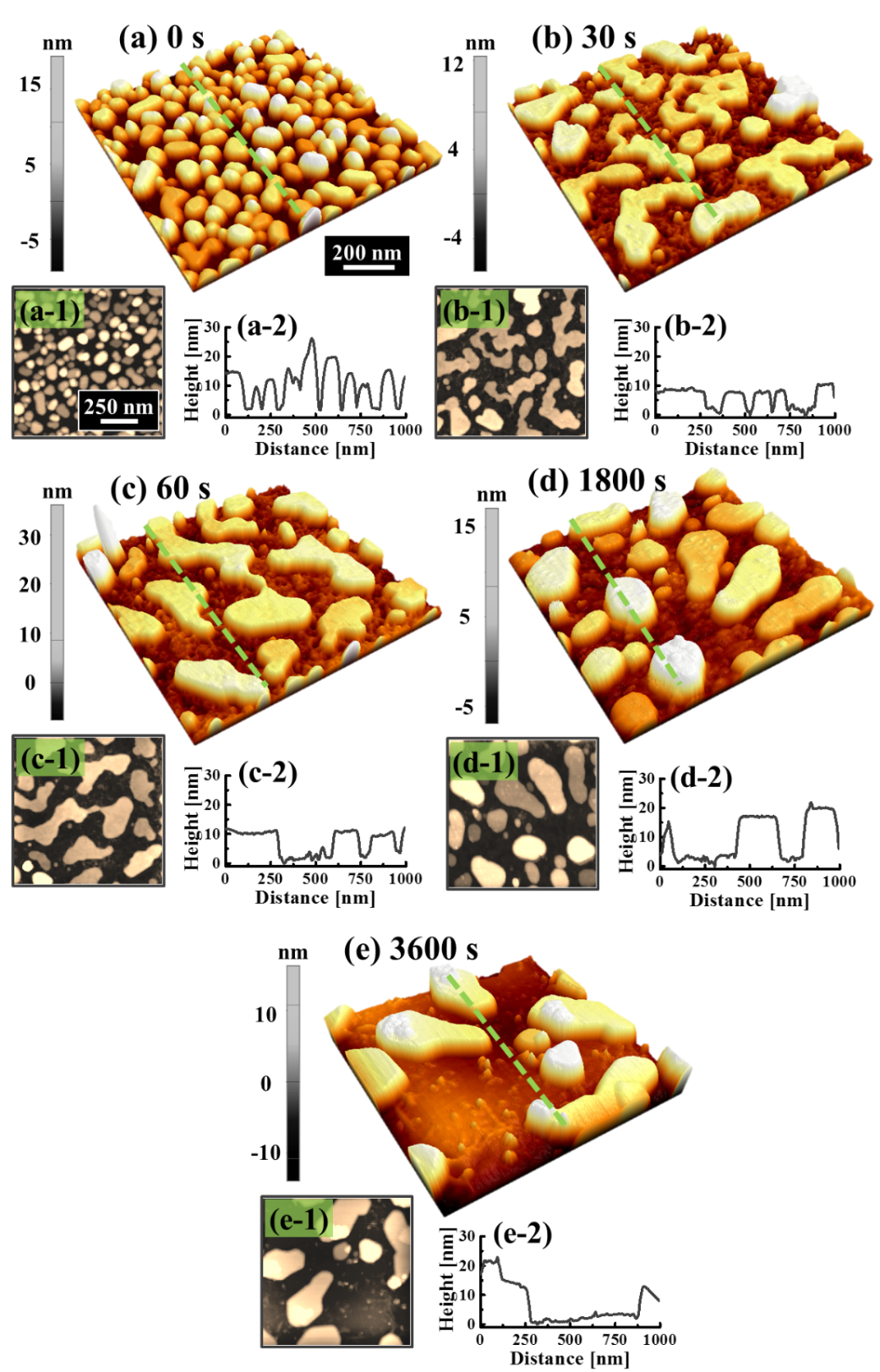
**

**S17 Fig.** (a) – (e) AFM side-views (1 × 1 µm^2^) of the Pt NPs formed by the annealing duration control from 0 to 3600 s at 800 ˚C with 15 nm Pt deposition thickness. (a-1) – (e-1) Corresponding top-views. (a-2) – (e-2) Cross-sectional line-profiles.
