# Supplementary material for: Effects of annealing temperature and duration on the morphological and optical evolution of self-assembled Pt nanostructures on c-plane sapphire
Source: PLoS One. 2017 May 4;12(5):e0177048. doi: 10.1371/journal.pone.0177048 (PMC5417639; doi:10.1371/journal.pone.0177048)
Supplement: S18 Fig — (a)–(f) Reflectance spectra of the Pt NPs on sapphire with variable annealing duration as labelled at 800°C and 15 nm initial Pt thickness. (g) Reflectance spectrum of bare sapphire. (h) Average reflectance with respect to the dwelling time. (i) Corresponding Raman spectra of A1g peaks. Summary plots of (j) A1g peak intensity, (k) Rq and (l) SAR. (DOCX) [file pone.0177048.s018.docx]

**
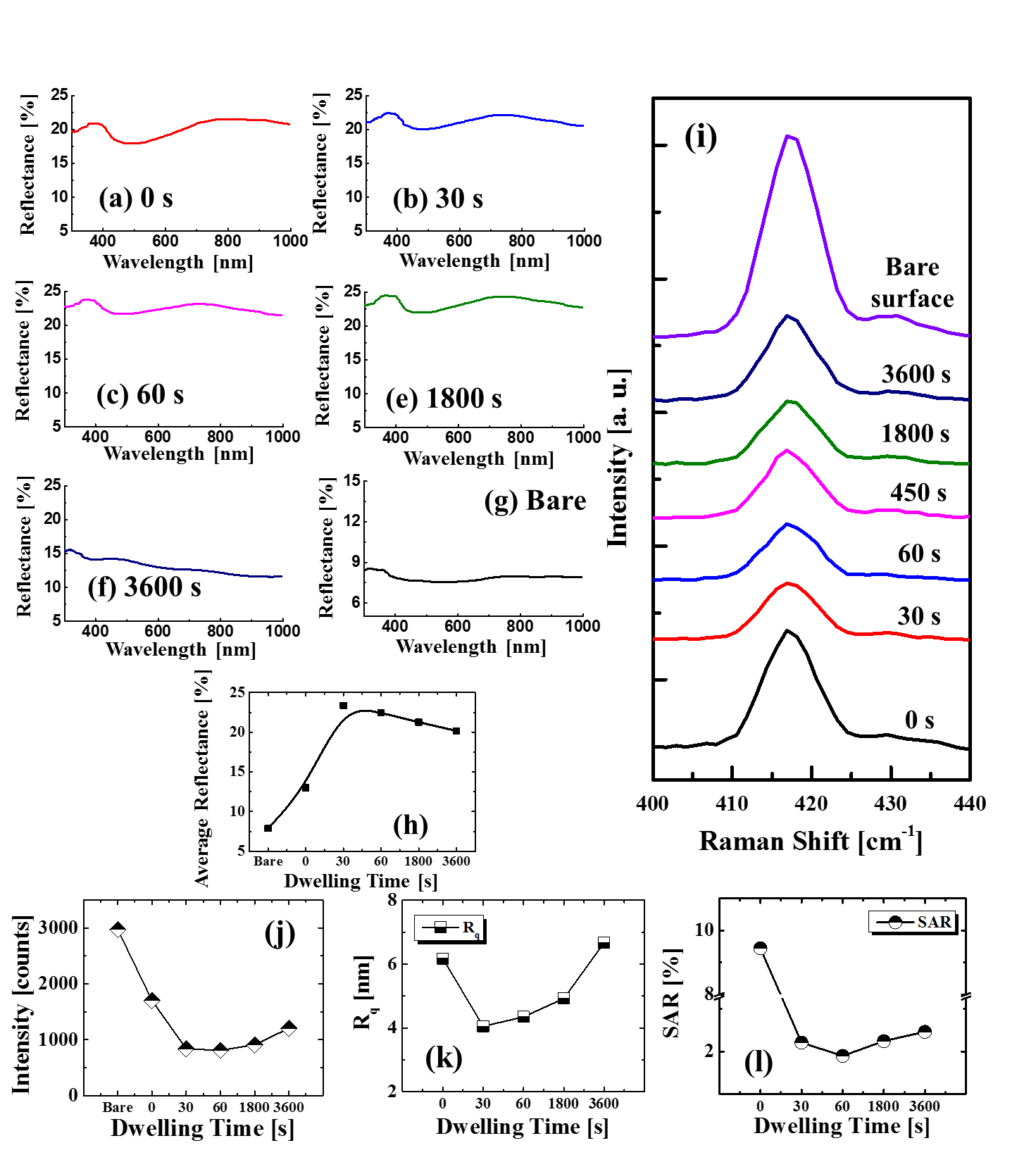
**

**S18 Fig.** (a) – (f) Reflectance spectra of the Pt NPs on sapphire with variable annealing duration as labelled at 800 ˚C and 15 nm initial Pt thickness. (g) Reflectance spectrum of bare sapphire. (h) Average reflectance with respect to the dwelling time. (i) Corresponding Raman spectra of A_1g_ peaks. Summary plots of (j) A_1g_ peak intensity, (k) Rq and (l) SAR.
