# Supplementary material for: Effects of annealing temperature and duration on the morphological and optical evolution of self-assembled Pt nanostructures on c-plane sapphire
Source: PLoS One. 2017 May 4;12(5):e0177048. doi: 10.1371/journal.pone.0177048 (PMC5417639; doi:10.1371/journal.pone.0177048)
Supplement: S1 Table — (DOCX) [file pone.0177048.s019.docx]

**S1 Table**. Summary of the root mean squared roughness (Rq) and surface area ratio (SAR) with the various deposition amounts of Pt (pre-anneal).

| **DA**  **[nm]** | **Rq**  **[nm]** | **SAR**  **[%]** |
| --- | --- | --- |
|  |  |  |
| **0 (Bare)** | 0.2 | 0.02 |
| **3** | 0.3 | 0.04 |
| **10** | 0.9 | 0.71 |
| **15** | 1.1 | 1.22 |
| **20** | 1.2 | 1.25 |
