# Supplementary material for: Effects of annealing temperature and duration on the morphological and optical evolution of self-assembled Pt nanostructures on c-plane sapphire
Source: PLoS One. 2017 May 4;12(5):e0177048. doi: 10.1371/journal.pone.0177048 (PMC5417639; doi:10.1371/journal.pone.0177048)
Supplement: S2 Table — (DOCX) [file pone.0177048.s020.docx]

**S2 Table**. Summary of Rq and SAR of the Pt nanostructures on sapphire with 3, 10, and 20 nm Pt deposition (DA) after annealing between 500 and 950 ˚C for 450 s.

| **DA**  **AT** | **3 nm** | | **10 nm** | | **20 nm** | |
| --- | --- | --- | --- | --- | --- | --- |
|  |  |  |  |  |  |  |
|  | **Rq [nm]** | **SAR [%]** | **Rq [nm]** | **SAR [%]** | **Rq [nm]** | **SAR [%]** |
| **500** | 0.30 | 0.08 | 0.20 | 0.03 | 0.40 | 0.11 |
| **550** | 0.60 | 0.32 | 1.20 | 0.12 | 0.50 | 0.11 |
| **600** | 0.70 | 0.52 | 2.00 | 0.37 | 0.70 | 0.27 |
| **650** | 0.70 | 0.50 | 3.50 | 1.83 | 1.10 | 0.45 |
| **700** | 0.70 | 0.41 | 3.00 | 1.21 | 2.30 | 0.75 |
| **750** | 0.70 | 0.45 | 2.60 | 0.80 | 4.20 | 1.30 |
| **800** | 0.70 | 0.39 | 2.80 | 1.35 | 6.10 | 1.92 |
| **850** | 0.70 | 0.32 | 3.90 | 1.98 | 6.00 | 1.41 |
| **900** | 0.70 | 0.30 | 4.20 | 1.97 | 7.40 | 1.66 |
| **950** | 1.00 | 0.75 | 4.50 | 4.10 | 9.20 | 1.98 |
