# Supplementary material for: Effects of annealing temperature and duration on the morphological and optical evolution of self-assembled Pt nanostructures on c-plane sapphire
Source: PLoS One. 2017 May 4;12(5):e0177048. doi: 10.1371/journal.pone.0177048 (PMC5417639; doi:10.1371/journal.pone.0177048)
Supplement: S3 Table — (DOCX) [file pone.0177048.s021.docx]

**S3 Table**. Summary of Rq and SAR of Pt nanostructures on sapphire with the dwelling time (DT) control between 0 and 3600 s having different initial thickness of Pt film.

| **DA**  **DT** | **15 nm** | | **20 nm** | |
| --- | --- | --- | --- | --- |
|  |  |  |  |  |
|  | **Rq [nm]** | **SAR [%]** | **Rq [nm]** | **SAR [%]** |
| **0** | 9.45 | 6.20 | 9.10 | 9.00 |
| **30** | 2.21 | 4.10 | 1.72 | 5.50 |
| **60** | 1.89 | 4.30 | 1.86 | 5.90 |
| **450** | - | - | 1.92 | 6.10 |
| **1800** | 2.24 | 4.90 | 2.15 | 7.40 |
| **3600** | 2.46 | 6.70 | 2.21 | 10.50 |
