# Supplementary material for: Effects of annealing temperature and duration on the morphological and optical evolution of self-assembled Pt nanostructures on c-plane sapphire
Source: PLoS One. 2017 May 4;12(5):e0177048. doi: 10.1371/journal.pone.0177048 (PMC5417639; doi:10.1371/journal.pone.0177048)
Supplement: S4 Table — Samples were fabricated with a variation of annealing temperatures (ATs) between 500 and 950°C. (DOCX) [file pone.0177048.s022.docx]

**S4 Table**. Summary of Raman intensity of Pt nanostructures on sapphire for the annealing temperatures (AT) control sets (10 and 20 nm). Samples were fabricated with a variation of annealing temperatures (ATs) between 500 and 950 ˚C.

| **DA**  **AT** | **10 nm** | **20 nm** |
| --- | --- | --- |
|  |  |  |
| **Bare** | 2236 | 2213 |
| **500** | 1368 | 476 |
| **550** | 1443 | 524 |
| **600** | 1343 | 516 |
| **650** | 1192 | 527 |
| **700** | 1025 | 482 |
| **750** | 1126 | 503 |
| **800** | 1080 | 614 |
| **850** | 1077 | 678 |
| **900** | 1408 | 744 |
| **950** | 2236 | 1071 |
