# Supplementary material for: Effects of annealing temperature and duration on the morphological and optical evolution of self-assembled Pt nanostructures on c-plane sapphire
Source: PLoS One. 2017 May 4;12(5):e0177048. doi: 10.1371/journal.pone.0177048 (PMC5417639; doi:10.1371/journal.pone.0177048)
Supplement: S5 Table — Samples were fabricated by the control of (DT) between 0 and 3600 s at a fixed annealing temperature (AT) of 800°C. (DOCX) [file pone.0177048.s023.docx]

**S5 Table**. Summary of Raman intensity of the Pt nanostructures on sapphire for the dwelling time (DT) control sets (15 and 20 nm). Samples were fabricated by the control of (DT) between 0 and 3600 s at a fixed annealing temperature (AT) of 800 ˚C.

| **DA**  **DT** | **15** | **20** |
| --- | --- | --- |
|  |  |  |
| **Bare** | 2972 | 2971 |
| **0** | 1702 | 1457 |
| **30** | 837 | 809 |
| **60** | 811 | 816 |
| **450** | - | 877 |
| **1800** | 911 | 1054 |
| **3600** | 1205 | 1190 |
